# Growth Behavior of Human Adipose Tissue-Derived Stromal/Stem Cells at Small Scale: Numerical and Experimental Investigations

**DOI:** 10.3390/bioengineering5040106

**Published:** 2018-12-04

**Authors:** Valentin Jossen, Regine Eibl, Matthias Kraume, Dieter Eibl

**Affiliations:** 1 Institute of Chemistry and Biotechnology, Zurich University of Applied Sciences, 8820 Wädenswil, Switzerland; regine.eibl@zhaw.ch (R.E.); dieter.eibl@zhaw.ch (D.E.); 2 Department of Process Engineering, Technical University of Berlin, 10623 Berlin, Germany, matthias.kraume@tu-berlin.de

**Keywords:** computational fluid dynamics, human adipose tissue-derived stromal/stem cells, humane telomerase reversed transcriptase immortalized hASCs, microcarrier, segregated growth model, Euler–Euler and Euler–Lagrange approaches, particle image velocimetry/shadowgraphy measurements

## Abstract

Human adipose tissue-derived stromal/stem cells (hASCs) are a valuable source of cells for clinical applications, especially in the field of regenerative medicine. Therefore, it comes as no surprise that the interest in hASCs has greatly increased over the last decade. However, in order to use hASCs in clinically relevant numbers, in vitro expansion is required. Single-use stirred bioreactors in combination with microcarriers (MCs) have shown themselves to be suitable systems for this task. However, hASCs tend to be less robust, and thus, more shear sensitive than conventional production cell lines for therapeutic antibodies and vaccines (e.g., Chinese Hamster Ovary cells CHO, Baby Hamster Kidney cells BHK), for which these bioreactors were originally designed. Hence, the goal of this study was to investigate the influence of different shear stress levels on the growth of humane telomerase reversed transcriptase immortalized hASCs (hTERT-ASC) and aggregate formation in stirred single-use systems at the mL scale: the 125 mL (=SP100) and the 500 mL (=SP300) disposable Corning^®^ spinner flask. Computational fluid dynamics (CFD) simulations based on an Euler–Euler and Euler–Lagrange approach were performed to predict the hydrodynamic stresses (0.06–0.87 Pa), the residence times (0.4–7.3 s), and the circulation times (1.6–16.6 s) of the MCs in different shear zones for different impeller speeds and the suspension criteria (*Ns1u, Ns1*). The numerical findings were linked to experimental data from cultivations studies to develop, for the first time, an unstructured, segregated mathematical growth model for hTERT-ASCs. While the 125 mL spinner flask with 100 mL working volume (SP100) provided up to 1.68 × 10^5^ hTERT-ASC/cm^2^ (=0.63 × 10^6^ living hTERT-ASCs/mL, EF 56) within eight days, the peak living cell density of the 500 mL spinner flask with 300 mL working volume (SP300) was 2.46 × 10^5^ hTERT-ASC/cm^2^ (=0.88 × 10^6^ hTERT-ASCs/mL, EF 81) and was achieved on day eight. Optimal cultivation conditions were found for *Ns1u* < *N* < *Ns1*, which corresponded to specific power inputs of 0.3–1.1 W/m^3^. The established growth model delivered reliable predictions for cell growth on the MCs with an accuracy of 76–96% for both investigated spinner flask types.

## 1. Introduction

Cell therapeutics and stem cell-based therapies in particular are viewed as clinical applications that will cause a healthcare revolution. Over the last years, various clinical trials with different stem cell types (e.g., human embryonic stem cells, human mesenchymal stem cells hMSCs, hematopoietic stem/progenitor cells) have demonstrated their clinical potential, efficiency, and safety [1,2,3,4]. From this large number of clinical trials, it is striking that hMSCs are the predominant stem cell type used in regenerative medicine (e.g., cardiology, immunology, neurology, and orthopedics) [3,5,6]. At the beginning of June 2018, as many as 253 clinical trials involving hMSCs were registered (www.clinicaltrials.gov). This is unsurprising due to their high proliferation potential, their immunosuppressive, immunoregulating, migrating, and trophic properties and low ethical concerns [6]. Their immunomodulatory properties also make hMSCs attractive for allogeneic therapies, which seem to be the most commercially attractive option at present [7,8]. However, only 13 hMSC-based products have received regulatory approval to date [9,10]. Nevertheless, it is speculated that this number will increase significantly over the coming years. Currently applied cell doses are in the range of 1 to 5 million hMSCs/kg body weight (=70–350 million hMSCs for a 70 kg person), demonstrating the need for safe and efficient in vitro expansion [9,11].

Numerous reports have shown that traditional, planar, and static cultivation systems such as stacked-plate systems are not well-suited to meeting the high cell numbers required by the current and future cell therapeutic market in an economical, reproducible, and safe way [8,12]. Hence, the hMSC production industry has been looking for alternatives for some time. The most attractive alternative to overcome the challenges of planar cultivation systems are instrumented, stirred bioreactors in combination with microcarriers (MCs). Special attention is being paid to single-use (also referred to as disposable) versions, which may significantly improve patient safety by reducing the risk of cross-contamination [12,13,14,15]. Even though single-use stirred bioreactors have recently been successfully used in various independent cultivation studies in which hMSCs were grown on MCs up to pilot scale [16,17,18], challenges still exist. While there are complex biological requirements of MCs (e.g., coating, substrate stiffness, etc.) and culture media (e.g., serum-free/substrate concentration, growth factors, etc.), these parameters also significantly affect the complexity of the culture process (=multiphase system with interactions between MC/MC, MC/cell, MC/impeller, cell/impeller, etc.). This is aggravated by the fact that commercially available stirred, single-use bioreactors were not originally designed for hMSC expansion processes. In fact, hMSCs are prone to higher shear sensitivity than conventional cell lines (e.g., CHO, BHK) that are grown in the majority of these systems. For this reason, it makes sense to fully characterize the systems of choice using appropriate biochemical engineering methods prior to system usage or during process development. Modern biochemical engineering tools such as CFD have proven themselves suitable for this purpose. For example, various reports have already described the potential of numerical fluid flow simulations to characterize stirred bioreactors for mammalian cell lines [19,20,21,22,23,24] and hMSCs [25,26,27,28,29,30,31,32,33]. However, the majority of the studies did not focus on the effects of hydrodynamic stresses on the cells and their growth. Moreover, aggregate formation in relation to cell growth has also received little attention.

Therefore, the aim of this study was to use multiphase CFD simulations (Euler–Euler, Euler–Lagrange) and other numerical and experimental methods to investigate the hydrodynamic behavior of MCs in two single-use spinner flasks with comparable geometrical ratios on a small scale and to derive optimal conditions for hMSC mass expansion and future scale-up. Fluid flow and particle-related parameters such as acting force, residence time, and circulation time were used to find general correlations for hydrodynamic parameters and cell growth. The biochemical engineering data were linked with growth-related parameters for hTERT-ASCs for the first time in order to establish a mathematical growth model for the MC-based expansion process. The hTERT-ASC cell line from the American Type Culture Collection (ATCC) was used in this study in order to ensure a robust cell expansion process (w/o the adverse effect of the in vitro senescence). This allows to determine the growth-related parameters, and finally, to establish a reliable growth model.

## 2. Materials and Methods

### 2.1. Bioreactor Systems: 125 mL and 500 mL Disposable Conring^®^ Spinner Flasks

**Figure 1:**
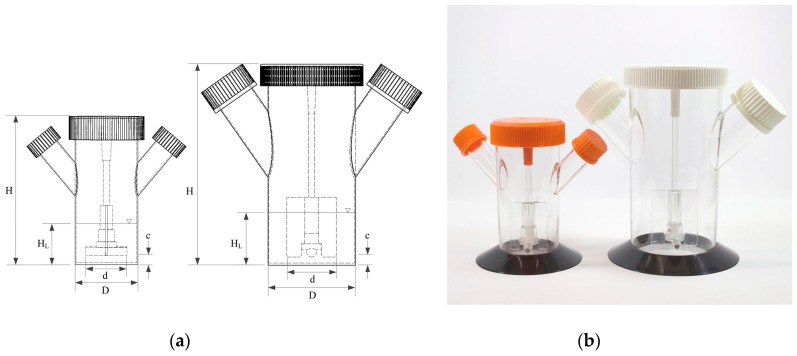
Small scale single-use Corning^®^ spinner flasks (125 mL and 500 mL). (**a**) Technical drawings with the main geometrical dimensions. (**b**) Picture of the spinner flasks.

Disposable Corning^®^ spinner flasks (Corning, Corning, NY, USA), which are commercially available in different sizes (125 mL and 500 mL, see Figure 1), were used for all investigations in this study.

These rigid culture containers are made of polycarbonate and were equipped with two angled side ports. The slightly opened lids provided gas exchange (CO_2_/O_2_) for surface aeration in a standard cell culture incubator during the cultivations. The main physical dimensions and ratios of the spinner flasks are summarized in Table 1. The maximum working volumes that were used for the cultivation studies were 100 mL (=SP100) and 300 mL (=SP300). Both spinner flasks were equipped, as standard, with paddle-like impellers consisting of a blade and a magnetic bar. As can be seen in Table 1, the used spinner flasks have comparable geometrical ratios.

### 2.2. Numerical Investigations

#### 2.2.1. CFD

For all CFD simulations, the fluid flow and the MC distribution were modeled using the Fluent 16.2 finite volume solver (ANSYS Inc., Canonsburg, PA, USA). The numerical technique was based on the subdivision of the fluid domain into a finite number of control volumes and the discretization of the time-averaged mass and momentum equations. This approach provided the algebraic equations, which were solved iteratively [19]. All simulations were run in parallel and solved on a computational cluster (up to 16 Intel^®^ Xeno^®^ E5-2630 v4 CPU’s @ 2.2 GHz, 64 GB RAM) in order to speed up the computational turnaround time.

**Euler–Euler (EE) approach:**

Multiphase simulations were carried out using the EE Reynolds-averaged Navier–Stokes (RANS) approach, which considered water (=culture medium) as the continuous and the MCs as the dispersed phase. The continuity equation for the *q^th^* phase was written as shown in Equation (1),

(1) $$\frac{\partial \left(\alpha_{q}\cdot \rho_{q}\right)}{\partial t}+\nabla \left(\alpha_{q}\cdot {\vec{u}}_{q}\cdot \rho_{q}\right)=0$$

where ${\vec{u}}_{q}$, $\rho_{q}$, $\alpha_{q}$ (*q* = *L*, the liquid phase and *q* = *P*, the particle phase) represented the phase velocity vector, the phase density and the phase volume fraction, respectively. The phases were assumed to share space in proportion to their volume fractions (see Equation (2)), whereas the maximum volume fraction of the dispersed phase was restricted to 0.63 (=maximum packing limit) due to the spherical shape of the MCs.

(2) $$\overset{n}{\underset{i=1}{\sum }}\alpha_{q}=1$$

The conservation equation for the momentum of the *q*th phase was based on an extended Navier–Stokes equation (see Equation (3)). The momentum exchange resulted in the coupling of the two phases.

(3) $$\frac{\partial \left(\alpha_{q}\cdot \rho_{q}\cdot {\vec{u}}_{q}\right)}{\partial t}+\nabla \left(\alpha_{q}\cdot \rho_{q}\cdot {\vec{u}}_{q}\cdot {\vec{u}}_{q}\right)+\alpha \nabla p-\nabla \left(\alpha_{q}\cdot {\bar{\tau }}_{q}\right)-\alpha_{q}\cdot \rho_{q}\cdot g_{q}+{\vec{F}}_{q}=0$$

In addition to all of the forces (i.e., viscous stresses, overall pressure gradient, gravitational force, interphase momentum forces) acting on a fluid element of the *q*th phase in the fluid domain, the drag force was considered to be the most important interphase force. In general, drag force results from the relative velocity between the two phases (see Equation (4)).

(4) $${\vec{F}}_{D}=\frac{3}{4}\rho_{L}\alpha_{L}\alpha_{P}\frac{C_{D}}{d_{P}}\left({\vec{u}}_{P}-{\vec{u}}_{L}\right)\left|{\vec{u}}_{P}-{\vec{u}}_{L}\right|$$

The drag coefficient *C_D_* was modeled in all of the EE simulations using the Symlal & O’Brien sub-model [34]. The Symlal and O’Brien sub-model was used due to numerical stability issues in the granular model. However, the computed *C_D_* values did not differ significantly from the expected *Re_P_* range compared to values derived from the standard correlation given by Schiller and Neumann [35], which was used for the Euler–Lagrange simulations (see Euler–Lagrange approach). The term $u_{r,p}$ in Equation (5) represents the terminal velocity correlation for the solid phase and is, therefore, dimensionless (see also References [34,36]).

(5) $$C_{D}={\left(0.63+\frac{4.8}{\sqrt{Re_{P}/u_{r,P}}}\right)}^{2}$$

**Euler–Lagrange (EL) approach:**

The EL model is suitable for dispersed flows, where the particles are non-homogeneously distributed. This model approach combines the description of the continuous phase (=culture medium) with a segregated description of the dispersed phase (=MCs) in the Lagargian frame. Each particle in the flow was characterized by its location (${\vec{\chi }}_{P}$), velocity (${\vec{u}}_{P}$), and other mechanical and thermodynamic variables, while the fluid phase was treated as a continuum by solving the Navier-Stokes equations. The computational effort for the EL model was higher than the EE model because of the separate treatment of each particle. Therefore, the number of particles in each simulation was set between 116,000 and 125,000 (phase fraction = 0.1–0.2%). The velocities of the dispersed phase were obtained by integrating the force balance on each individual particle. Consequently, this approach equates the particle inertia with forces acting on the particles and can be written as shown in Equation (6) (e.g., for the x-direction in Cartesian coordinates).

(6) $$\frac{d{\vec{u}}_{p}}{dt}={\vec{F}}_{D}\left({\vec{u}}_{L}-{\vec{u}}_{P}\right)+\frac{g_{x}\left(\rho_{P}-\rho_{L}\right)}{\rho_{P}}+{\vec{F}}_{x}$$

The term ${\vec{F}}_{\chi }$ denotes additional forces in the particle force balance, such as the Coriolis force, centrifugal force, virtual mass force, Saffman lift force, Basset force, Magnus force, and pressure gradient-dependent forces. As mentioned for the EE model, the drag force was considered to be the important interphase force. Therefore, the drag force ${\vec{F}}_{D}$ was also introduced in the EL simulations. The drag coefficient *C_D_* was calculated using the standard correlation given by Schiller and Neumann [35] (see Equation (7)).

(7) $$C_{D}=\{\begin{array}{l} Re_{P}\leq 1000\;:\;\frac{24}{Re_{P}}\cdot \left(1+0.15\cdot Re_{P}^{0.687}\right)\\ Re_{P}>1000\;:0.44 \end{array}$$

**Numerical details and boundary conditions:**

The EE and EL simulations were carried out for maximum working volumes of either 100 mL (=SP100) or 300 mL (=SP300). In both cases, a hybrid mesh consisting of unstructured tetrahedral elements and a prism layer at the vessel walls was used. The meshes were generated using the ANSYS Meshing Tool, which was implemented in ANSYS Workbench 16.2 (ANSYS Inc., Canonsburg, PA, USA). A previous grid sensitivity study had confirmed that grids used with 710,000 (SP100) and 2,100,000 (SP300) fluid elements result in grid-independent results (data not shown). For both spinner flasks, the vessel and impeller walls were treated as non-slip boundaries with standard wall functions. The impeller rotation was implemented using the sliding mesh approach and the turbulence was modelled using the k-ω SST or k-ε realizable turbulence models, depending on the expected Re range. All multiphase simulations were performed transiently (dt = time corresponding to 1–3° of impeller motion) and simulation convergence within a time step was assumed when the residuals dropped below 10^−5^. The simulated time corresponded to at least three times the mixing time ($\Theta_{95\%}$) of the individual spinner flask (SP100 $\Theta_{95\%}$ = 7–17 s/25–120 rpm; SP300 $\Theta_{95\%}$ = 6–17 s/20–100 rpm) at the defined impeller speed. The phase-coupled SIMPLE algorithm (Semi-implicit Method for Pressure Linked Equations) [36] was used for pressure-velocity coupling in all cases.

#### 2.2.2. Segregated Growth Model (SGM)

**Figure 2:**
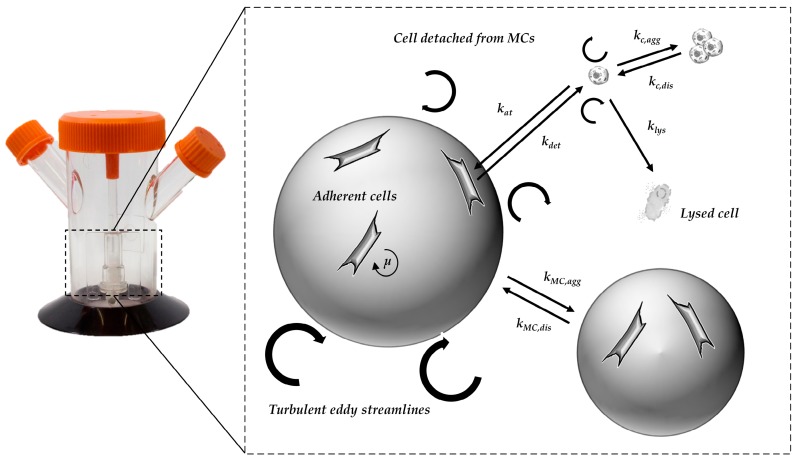
Principle of growth model and influencing factors.

Based on the findings from the CFD simulations (e.g., P/V, *τ_nt_*) and their combination with the cell culture data, an unstructured, segregated, simplistic growth model was developed. The general concept of the growth model and the influencing factors are shown in Figure 2. Because hMSC growth is anchorage-dependent, the cell aggregation and the formation of spheroids in the suspension was ignored. It was assumed, that cells in suspension do not contribute to the increase in overall cell numbers, with cell growth restricted to the MC surface. However, cells in suspension do affect the glucose balance, since they still have a maintenance metabolism. To define the starting conditions, it was assumed that initial cell attachment took place during the cell attachment phase. The cell attachment rate was described by the constant *k_at_* (see Table 2). Once the cells had attached to the MC surface, it was assumed that they immediately start to proliferate.

The specific cell growth rate (μ) was calculated based on Monod-type kinetics, with the consumed substrate (*Glc*), the produced metabolites (*Lac*, *Amn*), and the available growth surface (*X_max_*) considered as influencing factors (see Equation (8)). A comparable approach was already successfully used by Möhler et al. [37] and Bock et al. [38] to simulate anchorage-dependent Madin–Darby Canine Kidney (MDCK) cell growth in a MC-based culture.

(8) $$\mu =\mu_{max}\left(\frac{Glc}{K_{Glc}+Glc}\right)\cdot \left(\frac{K_{Lac}}{K_{Lac}+Lac}\right)\cdot \left(\frac{K_{Amn}}{K_{Amn}+Amn}\right)\cdot \left(\frac{X_{max}-X_{MC}}{X_{max}}\right)$$

The concentration of the cells on the MC surface increased through mitotic cell division and the attachment of cells from the suspension (see Equation (9)). However, this increase was reduced by cell detachment from the MC surface and was accounted for by the detachment constant −*k_det_*.

(9) $$\frac{dX_{MC}}{dt}=\mu \cdot X_{MC}+k_{at}\frac{X_{max}-X_{MC}}{X_{max}}X_{Sus}-k_{det}\cdot X_{MC}$$

However, the detachment constant −*k_det_* is strongly affected by hydrodynamic forces, and therefore, variable for different specific power inputs. As mentioned before, cell growth in the suspension was negligible and therefore changes in cell concentration will only be affected by cell attachment to or detachment from the MC surface (see Equation (10)).

(10) $$\frac{dX_{Sus}}{dt}=k_{det}\cdot X_{MC}-k_{at}\frac{X_{max}-X_{MC}}{X_{max}}X_{Sus}$$

Contrary to the growth restriction based on the specific growth rate, glucose consumption was only limited by the glucose concentration itself (see Equation (11)). Consequently, glucose consumption was the result of the glucose uptake by the mitotic cells and the maintenance metabolism of the mitotic and non-mitotic cells (*X_v_*).

(11) $$\begin{array}{c} \frac{dGlc}{dt}=-\frac{1}{Y_{\frac{X}{Glc}}}\mu_{max}\frac{X_{max}-X_{MC}}{X_{MC}}\frac{Glc}{K_{Glc}+Glc}\frac{K_{Lac}}{K_{Lac}+Lac}\frac{K_{Amn}}{K_{Amn}+Amn}X_{MC}-m_{Glc}\\ \;\cdot \sigma \left(Glc\right)\cdot X_{V} \end{array}$$

In order to avoid negative glucose concentrations, a step response was implemented (see Equation (12)).

(12) $$\sigma \left(Glc\right)=\{\begin{array}{l} 1\;\mathrm{for}\;Glc>0\\ 0\;\mathrm{for}\;Glc=0 \end{array}$$

Due to stability issues in the culture medium, ultra-glutamine (alanyl-L-glutamine) was used in all of the cultivation studies. However, glutamine consumption was not considered in the growth model, since our previous cultivation studies have shown that glutamine (*Gln*) is not a limiting factor in the medium, especially under the investigated conditions (data not shown). The production of the two main metabolites (*Lac*, *Amn*) was accounted for by a growth associated assumption, i.e., they were only produced during cell growth when sufficient substrate concentration and free growth surface were available (see Equations (13) and (14)). This meant that any increase in concentration due to maintenance metabolism was ignored.

(13) $$\frac{dLac}{dt}=q_{Lac}\cdot \mu \cdot X_{MC}$$

(14) $$\frac{dAmn}{dt}=q_{Amn}\cdot \mu \cdot X_{MC}$$

All calculations and simulations were performed with MATLAB R2018a (MathWorks Inc., Natick, MA, USA). The model equations were solved using the *ode15s* solver.

### 2.3. Biochemical Engineering Investigations

#### 2.3.1. Suspension Studies

The suspension criteria (*N_s1u_*, *N_s1_*) for the ProNectin^®^ F-COATED MCs ($\rho_{P}$ 1026 ± 4 kg/m^3^, $d_{P}$ 169 ± 43 μm, $A_{P}$ 360 cm^2^/g) were determined in both spinner flasks. The methodology for the determining of the suspension criteria was in accordance with Kaiser et al. [25]. In brief, the *N_s1_* (=*N_js_* or just suspended) suspension criterion was defined as the impeller speed required to just fully suspend the MCs in the spinner flasks [40]. *N_s1u_* described the suspension state at which some of the MCs were still in contact with the reactor bottom, but none of them were at rest [41]. The suspension experiments were carried out at different MC concentrations (2.5–20 g/L) and with a specially developed cell culture medium from Lonza (w/5% FBS). While the impeller was in motion, the suspension state was recorded by two digital cameras (from the side and below) and the recordings were subsequently evaluated by visual observation. The optical accessibility to the spinner flask bottom was improved by a mirror which was placed below the flask.

#### 2.3.2. Particle Image Velocimetry (PIV)

**Figure 3:**
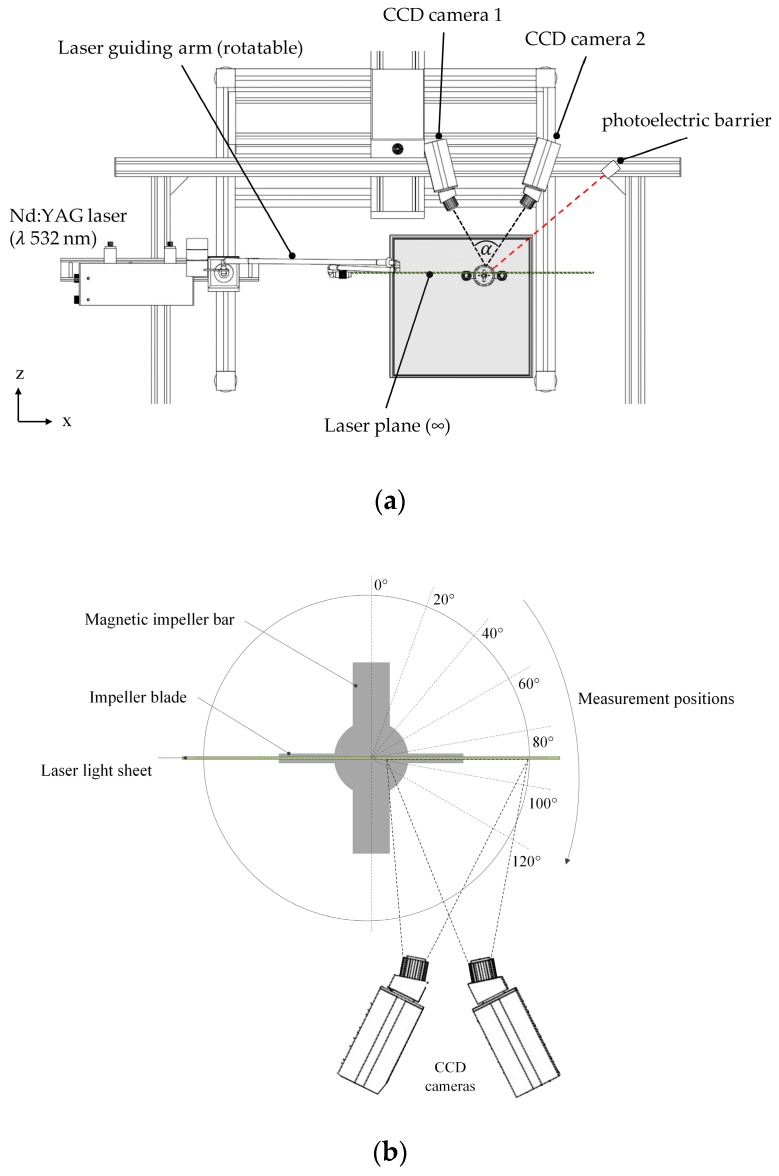
Measurement set-up for stereoscopic particle image velocimetry (PIV) measurements. (**a**) Schematic representation of the measurement setup from the top. (**b**) Orientation of the impeller and the laser light sheet plane during the measurements.

Stereoscopic PIV measurements were carried out using a FlowMaster PIV system (LaVision, Göttingen, Germany) in order to verify the CFD simulation results. The illumination of the field of investigation was performed by a double-pulsed Nd:YAG laser (Litron Laser Ltd., Rugby, UK), which generated a laser light sheet at a wavelength of λ 532 nm. In both cases, the laser light sheet was vertically aligned through the spinner flasks and allowed radial, axial, and tangential fluid velocity components to be measured (see Figure 3a). The fluid flow field was measured at different impeller positions by phase-resolved measurements using a photoelectric barrier (see Figure 3b). Two Imager Pro X4 CCD cameras (LaVision, Göttingen, Germany) with a resolution of 2048 × 2048 pixels were used for image capturing. A *Scheimpflug* set-up with backward/forward scattering was used to align the cameras, which were spatially calibrated using a two-level calibration plate (106-10, LaVision, Germany). In order to reduce the effect of refraction/diffraction, which was induced by the laser light beam hitting the cylindrical spinner flask surface, the spinner flasks were placed in a water-filled cubical box. The DaVis 8.3 (v 8.3, LaVision, Göttingen, Germany) software was used for image acquisition and image processing, with up to 1,500 double-frame images per camera considered for analysis by cross correlation. Cross correlation was performed by means of 32 × 32 pixel-interrogation windows and an overlap of 50%. Fluid flow visualization was achieved by adding rhodamine coated polymethylmethacrylat beads (PMMA, 1190 kg m^−3^, 20–50 μm, λ_EM,max_ 584 nm) to the spinner flasks. Finally, laser light reflections were reduced by means of corresponding long pass optical fluorescence edge filters (100% transmission >545 nm) mounted on each CCD camera.

#### 2.3.3. Microcarrier Measurement by Shadow Imaging (Shadowgraphy)

**Figure 4:**
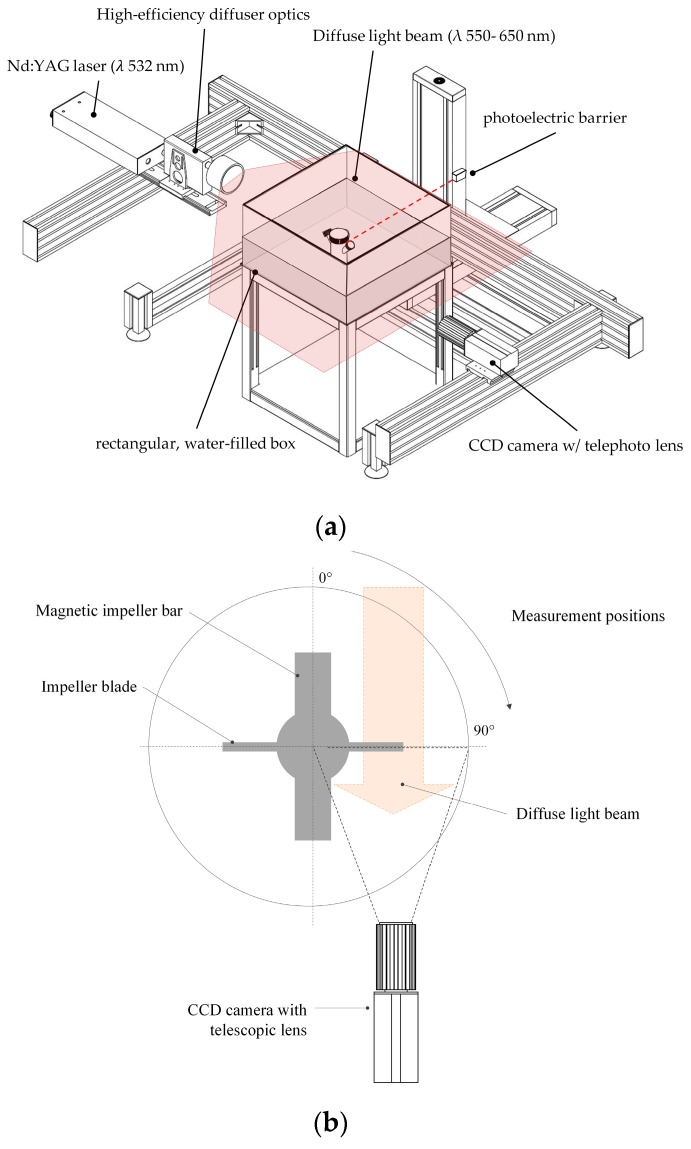
Measurement setup for shadow imaging investigations (Shadowgraphy). (**a**) Schematic representation of the measurement setup from the top. (**b**) Orientation of the impeller and the diffuse light beam during the measurements.

To verify the predicted MC characteristics from the multiphase simulations, MC distribution and velocities were measured experimentally by means of shadow imaging techniques (see Figure 4a). For this purpose, the ParticleMaster shadowgraphy system (LaVision, Göttingen, Germany) in conjunction with a double-pulsed Nd:YAG laser and a high-efficiency light diffusor (λ 550–600 nm) were used. The light beam was aligned parallel to the impeller shaft and directly opposite the CCD camera (Imager Pro X 4M, LaVision, Göttingen, Germany), which was equipped with a telephoto lens (Nikon, Tokyo, Japan). Similar to the PIV measurements, the spinner flasks were placed in a water-filled cubical box to reduce the effects of refraction/diffraction. DaVis 8.3 software (v 8.3, LaVision, Göttingen, Germany) and ParticleMaster toolbox were used for image acquisition and analysis. Particle recognition was performed based on an image segmentation algorithm with subsequent analysis of the pixel intensity profile [42]. To calculate the MC velocities, individual particles were tracked over two images and the velocities were calculated based on the corresponding pixel shift in the x–y directions (*v_xy_*). Since only one CCD camera was used for the investigations, the direct measurement and calculation of $\vec{w}$ was not possible. However, the effects of the particle shift in the z-direction were considered by means of depth-of-field calibration. Depending on the size of the spinner flask, 1,500 double-frame images were captured from up to 4 positions in the vertical direction. Spatial scanning of the fluid domain in the vertical direction was performed by a traverse system. All measurements were carried out with cell-free and cell-loaded MCs at different impeller speeds (*N_s1u_*/*N_s1_*) and impeller positions (see Figure 4b). The cell-loaded measurements were immediately performed at the end of 7 days of cultivating hTERT-ASCs. Synchronization of the camera and the laser to the impeller motion was performed by means of a trigger signal obtained from a photoelectric barrier in order to perform phase-resolved measurements.

### 2.4. Cultivation Studies

#### 2.4.1. Cells, Microcarriers, and Medium

Human telomerase reversed transcriptase immortalized hASCs (hTERT-ASCs) from the American Type Culture Collection (SCRC-4000^TM^, ATCC, Manassas, VA, USA) were used for all cultivation experiments. Prior to the cultivation studies, the hTERT-ASCs (*p* = 23, PDL 33) were adapted to the serum-reduced cell culture medium (5% FBS) from Lonza (data not shown) and a cryovial-based working cell bank was established (storage in liquid nitrogen). For the inoculation of each spinner flask, an initial average cell density of 3,000 cells/cm^2^ (corresponding to 10,800 cells/mL) and a MC concentration of 10 g/L (ProNectin^®^ F-COATED, Pall SoloHill, New York, NY, USA) was used. The MC concentration of 10 g/L was selected based on previous investigations of Schirmaier et al. [16]. The required number of MCs was prepared and sterilized as recommended by the vendor one day before inoculation.

#### 2.4.2. Analytics

**Figure 5:**
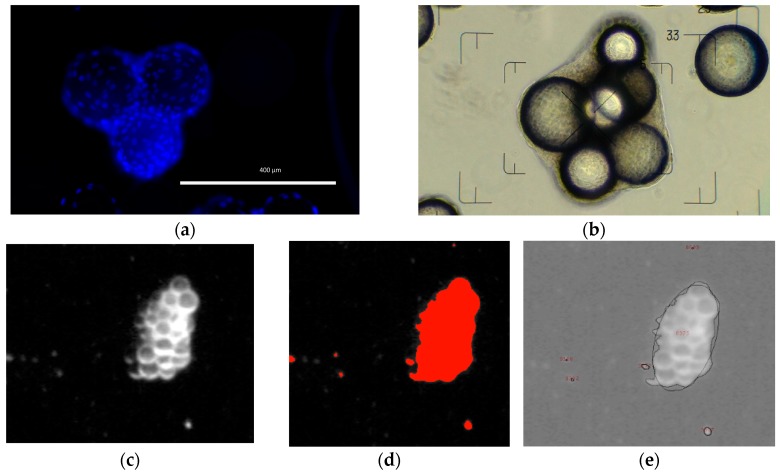
Image based analysis of MC-cell aggregates. (**a**) 4’,6-diamidin-2-phenyliondol (DAPI) stained picture. (**b**) Phase contrast microscopic picture for evaluation of aggregate size and shape. (**c**,**d**) Three step image processing for automated MC-cell aggregate analysis (I: image conversion, II: image segmentation, III: aggregate analysis).

Off-line samples were taken daily to measure the substrate (*Glc*, *Gln*) and metabolite (*Lac*, *Amn*) concentrations with a BioProfile 100Plus (Nova Biomedical, Waltham, MA, USA) and/or a CedexBio (Roche Diagnostics, Risch-Rotkreuz, Switzerland). After the cells had detached from the MC surface (15 min with TrypLE Select; Gibco by Life Technologies, Carlsbad, CA, USA), the hTERT-ASC cell number was measured (*n* = 3) using a NucleoCounter^®^ NC-200^TM^ (ChemoMetec, Allerød, Denmark). The measured cell specific values were used to calculate the growth-related parameters (maximum specific growth rate, $\mu_{max}$, minimum doubling time, *td*, expansion factor, *EF*). In addition to the cell measurements, 2 mL of the MC-cell suspension was fixed immediately after sampling with a 3% paraformaldehyde solution for 4’,6-diamidin-2-phenyliondol (in short DAPI) staining (see Figure 5a). The fixed MC-cell suspension was also used to analyze the MC-cell aggregates (see Figure 5b). For this purpose, the MCs were scanned prior to staining with a document scanner (Epson Perfection 1650) with an image resolution of 4,200 dpi (=5 µm/pixel). The images were then processed and analyzed with ImageJ (particle analysis toolbox) and Matlab in order to obtain the MC-cell aggregate size distribution (see Figure 5c–e). The number of cells in the culture supernatant (these are the cells detached from the MC surfaces) was measured daily by analyzing 0.4 mL of supernatant with a MACSQuant Analyzer 10 (Miltenyi Biotec, Bergisch Gladbach, Germany).

Flow cytometric measurements were carried out with cells from the inoculum and with microcarrier-free, purified cell samples from the different spinner flasks. For flow cytometric analysis, the cells were stained with fluorochrome-conjugated anti-human CD14, CD20, CD34, CD45, CD73, CD90, and CD105 (according to the recommendations of the International Society for Cellular Therapy ISCT and the International Federation for Adipose Therapeutics and Science IFATS) antibodies (Miltenyi Biotec, Germany) and measured with a MACSQuant device. Flow cytometric data were analyzed with Flowlogic (Inivai Inc., Mentone Vicoria, Australia) and presented as mean ±SD. A one-way ANOVA and a Holm–Sidak test (multiple comparisons versus control group) were used to compare numeric data amongst the different experimental groups. *p*-values less than 0.05 were accepted as statistically significant.

#### 2.4.3. Spinner Flask Cultivations

For each condition, three spinner flasks (*n* = 3) of both spinner flask types were inoculated with hTERT-ASCs and cultivated for 10 days at 37 °C, 5% CO_2_, and 80% humidity. All spinner flasks were inoculated with the same inoculum (P24, PDL 35), which was prepared using cryopreserved hTERT-ASCs (one passage post thawing in T_75_ flasks; 5000 cells/cm^2^). In addition, the cells were also expanded in a T75-flask in parallel as a static control. Prior to inoculation, the MC suspension was equilibrated for 12 h. Post inoculation, no agitation was performed for 4 h in order to support cell attachment on the MC surface. After the cell attachment phase, the impeller speed was set according to the individual experimental conditions (SP100 = 25 rpm, 49 rpm *N_s1u_*, 60 rpm *N_s1_*, 120 rpm; SP300 = 20 rpm, 41 rpm *N_s1u_*, 52 rpm *N_s1_*, 100 rpm). On days 4 and 8, partial medium exchanges (50%) were performed. For this purpose, the impellers were switched off and the MCs were allowed to settle. Fifty percent of the working volume of the spinner flasks was replaced with fresh preheated medium, and the impellers were restarted. No MC feeds were performed during the cultivations.

## 3. Results and Discussion

### 3.1. Suspension Studies

**Figure 6:**
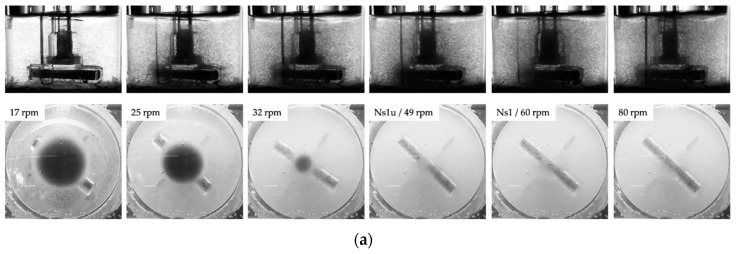

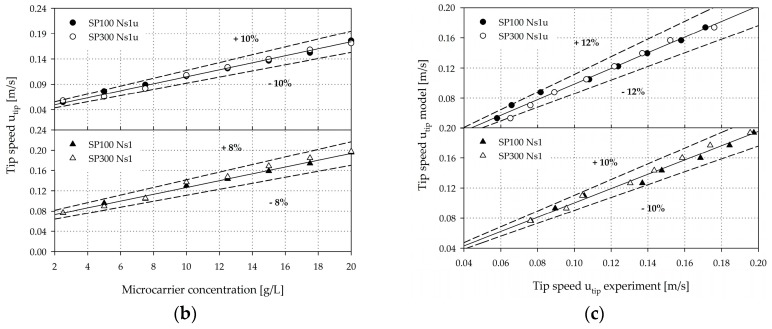
Microcarrier suspension dynamics. (**a**) Photographic pictures of the MC distribution during the suspension studies (e.g., SP100, 10 g/L). (**b**) Graphical representation of the required tip speeds to achieve the suspension criteria at different MC concentrations for the SP100 and SP300. (**c**) Experimental vs. predicted *Ns1u* and *Ns1* (=shown as tip speed) for the Corning spinner flasks (merged data for SP100 and SP300).

Different MC suspension states are represented in Figure 6a for the Corning^®^ SP100 with a MC concentration of 10 g/L. As can be seen from the images, different suspension states can be detected: (1) transport of the MCs to the vessel center and the formation of a clear outer zone, (2) swirling up of the MCs from the center of the vessel bottom and further reduction of the MC solid fraction at the reactor bottom, and (3) maintaining the MCs in suspension (*Ns1u* < *N* < *Ns1*). Comparable suspension states were also observed for the SP300 spinner flask. This was not surprising, since the two spinner flasks have comparable geometrical ratios and are equipped with identical impellers: a large paddle-like impeller (d/D 0.58–0.68). Due to the shape of the paddle-like impeller and the absence of probes, which probably act as baffles, a mainly tangentially oriented fluid flow was induced. The secondary flow resulted in the transport of the MCs to the vessel center (see Section 3.2). From this area, the MCs were swirled up as the impeller speed was further increased.

The impeller speeds required to fulfill the two suspension criteria for different MC concentrations (2.5–20 g/L) were in the range of 30 rpm to 81 rpm (*Ns1u*) and 35 rpm to 90 rpm (*Ns1*) for the SP100 and 22 rpm to 65 rpm (*Ns1u*) and 29 rpm to 75 rpm (*Ns1*) for the SP300. Therefore, it can be concluded that *Ns1* = (1.1–1.3)·*Ns1u*. Within the investigated MC concentration range, a linear correlation was found between the impeller speeds required to achieve the suspension criteria for the corresponding MC concentration. Thus, an average increase of 27 rpm per g MCs for the SP100 and an average increase of 29 rpm per g MCs for the SP300 are required to maintain the suspension state defined by *Ns1u* and *Ns1*. The impeller speeds required to ensure the suspension criteria in the SP100 were on average 25.25 ± 6.5% higher than for the SP300. However, when comparing the corresponding tip speeds (*u_tip_* = *π n d*), similar values for the SP100 and SP300 were calculated (see Figure 6b). This can be explained by the comparable geometrical ratios of the two systems and the resulting fluid flow conditions, which are described in Section 3.2. The maximum deviations of the measured data from the predicted values were between 8% and 10%, depending on the linear regression analysis for *Ns1u* (*u*_*tip*,*Ns1u*_ = 0.0067·c_MC_ + 0.0379) and *Ns1* (*u*_*tip*,*Ns1*_ = 0.0069·c_MC_ + 0.0581). However, these statistical correlations are only valid within the tested MC concentration range (2.5–20 g/L). The accuracy of the two regression models is also shown in Figure 6c, where the experimentally measured values were plotted against the predicted model values. It can be clearly seen that the values only slightly scatter around the diagonal center line, and the maximum deviations were between ±10% and ±12%. Nevertheless, the results demonstrate the linear relationship between the tip speed and the MC concentration as well as the applicability of the regression models for estimating the suspension criteria within the tested MC concentration range for the two spinner flasks. In fact, the dependency of the suspension criteria on the geometrical dimensions is undeniable. The determined impeller speeds and regression models served as a basis for the CFD simulations (see Section 3.2, Section 3.3, Section 3.4 and Section 3.5).

### 3.2. Single-Phase Fluid Flow Pattern

**Figure 7:**
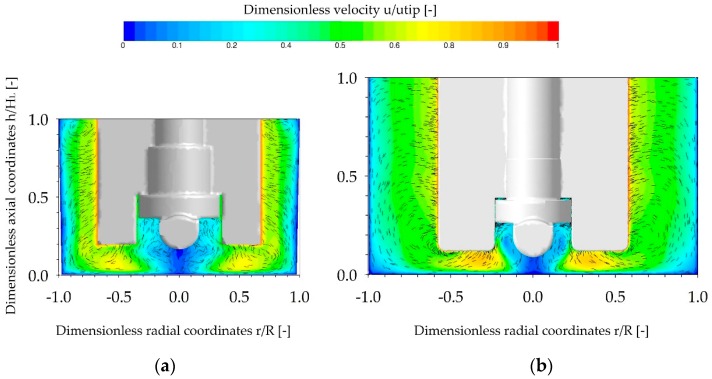
Fluid flow inside the SP100 (**a**) and SP300 (**b**), represented by combined vector and contour plots. The fluid flow pattern is presented in the vertical mid-plane for the *N_s1u_*-criterion (SP100 49 rpm, SP300 41 rpm). The velocities are normalized by *u*/*u_tip_* and the vectors are projected to the given plane with a fixed length of 0.1 mm.

The main objective of the single-phase CFD simulations was to characterize and compare the fluid flow under the same conditions investigated in the cultivation studies (see Section 3.5). As shown in Figure 7, the fluid flow profiles in the two spinner flask types were similar, due to their comparable geometrical ratios. In both cases, the highest fluid velocities occurred at the edges of the impeller blades and corresponded quite well to the theoretical tip speed. An area with relatively weak fluid velocities was generated directly below the impeller (*r*/*R* ± 0.3) in both systems. Thus, this area represents a critical zone for MC sedimentation. The observed MC transport form the outer part of the vessel to the vessel center was mainly driven by the induced secondary flow, previously discussed in Section 3.1. Similar findings were also reported by Berry et al. [31], Liovic et al. [28], and Venkat et al. [43] in other types of small scale spinner flasks.

**Figure 8:**
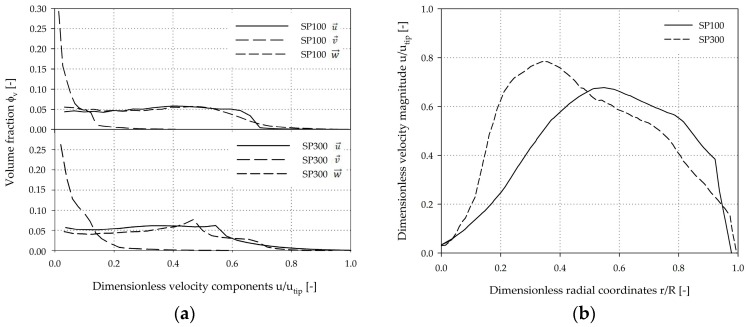
Comparison of fluid velocities at the experimentally determined *N_s1u_* criterion. (**a**) Volume-weighted fractions of the velocity components in x, y, z-directions. The volume-weighted data was discretized into 30 discrete classes and normalized by *u_tip_*. (**b**) Normalized velocity magnitude (*u*/*u_tip_*) along radial coordinates at c/2. $\vec{u},\;\vec{v},\;\vec{w}/u_{tip}$ = dimensionless velocities in x, y, z-directions.

A more quantitative comparison of the volume-weighted fractions of the individual velocity components (x, y, z-directions) is shown in Figure 8a. The profiles of the velocity components are very similar for both systems. The results indicate that the fluid flow in both systems was mainly tangentially oriented. As defined by the boundary conditions, maximum tangential fluid velocity of 0.99 *u_tip_* (SP100) and 1.0 *u_tip_* (SP300) were expected. It can be concluded that the velocity distribution is relatively homogeneous without large fluctuations in the volume-weighted velocity profile. The axial part of the fluid flow was not particularly pronounced and the maximum values were between 0.41 *u_tip_* (SP100) and 0.59 *u_tip_* (SP300). The highest axial fluid velocities occurred directly below the impeller blade and were crucial for MC dispersion. The comparison of the dimensionless velocity magnitude (*u*/*u_tip_*) along the dimensionless radial coordinates (*r*/*R*) at c/2 (=4 mm) indicated a greater velocity increase and a higher maximum fluid velocity in the SP300, which can be ascribed to the wider impeller blade (≈20 mm) (Figure 8b). However, the spatial dimensions of the critical fluid zone have similar courses in both spinner flasks.

### 3.3. Fluid Flow Field Verification

**Figure 9:**
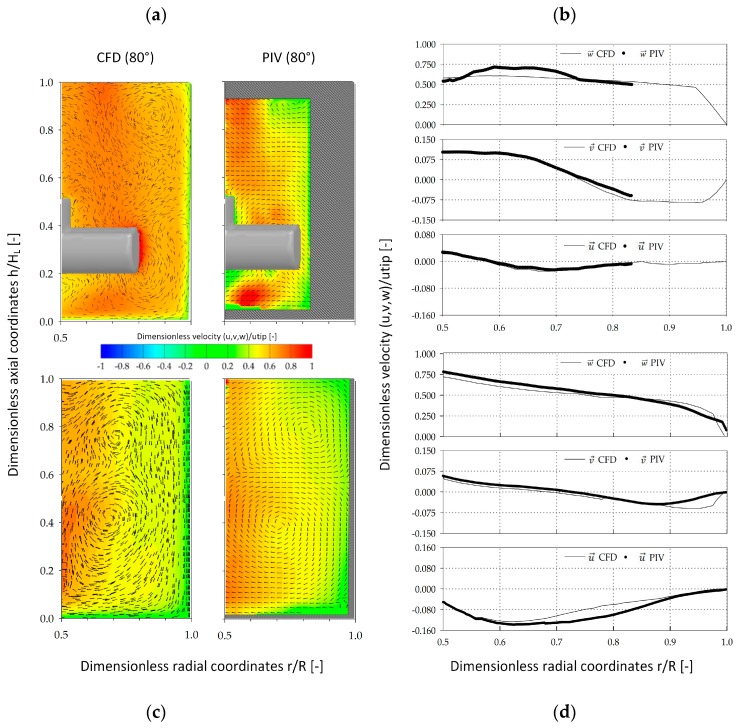
Verification of the fluid flow pattern in the SP100 and SP300. Qualitative (**a**,**c**) and quantitative (**b**,**d**) comparison of CFD-predicted and PIV-measured fluid velocity components ($\vec{u},\vec{v},\vec{w}$) in the SP100 (**a**,**b**) and SP300 (**c**,**d**). The contour plots are colored according to $\vec{w}$. The arrows show the vectors in the x and y-directions. The quantitative comparison was performed along radial coordinates at a dimensionless axial coordinate of 0.1 (h/H_L_).

Since a number of mathematical assumptions were used for the CFD modeling of the two spinner flasks, stereoscopic PIV measurements were performed to validate the CFD-predicted fluid flow pattern (Figure 9). The contour plots show the fluid flow vectors in the x and y-directions as well as the fluid velocity component $\vec{w}$ at a spatial position of 80° along mid plane of the vessel. A comparison of the fluid velocities in the SP100 was only possible for dimensionless radial coordinates between 0.50 and 0.82 (*r*/*R*), due to the pronounced curve of the vessel surface. Nevertheless, the qualitative comparison of the fluid velocity vectors showed good agreement between the CFD model and the PIV measurements. The only differences were slightly underestimated fluid velocities (0.76 *u_tip_*) that were determined near the impeller bar. These differences can be accounted for by measurement uncertainties based on optical phenomena and the restricted measurement accuracy directly at the edges of the impeller bar. Thus, direct comparison of the fluid velocities in direct proximity to the impeller is difficult. The qualitative comparison of the fluid flow pattern in the SP300 showed very good matching between the CFD-predicted and the actual fluid flow structures, with two recirculating flows. For a more quantitative comparison of the individual velocity components, the CFD-predicted and PIV-measured data were compared along dimensionless radial coordinates (0.5–1.0 *r*/*R*) at an axial position of 0.1 (h/H_L_). The comparison of the CFD-predicted and PIV-measured velocity components in the SP100 revealed only minor differences for $\vec{v}$ (up to 7.5%) and $\vec{w}$ (up to 8.7%). However, the CFD velocity profiles were well captured and the overall agreement of PIV and CFD was satisfactory, findings are consistent with those published by Kaiser et al. [25]. All three velocity components in the SP300 were well captured by the stereoscopic PIV-measurements. The greatest differences (7.9–15%) were found for $\vec{u}$ between 0.7 and 0.85 (*r*/*R*). Hence, it can be postulated that, regardless of the differences, the established CFD model provides reliable fluid flow predictions in both spinner flask types.

### 3.4. Microcarrier Distribution

**Figure 10:**
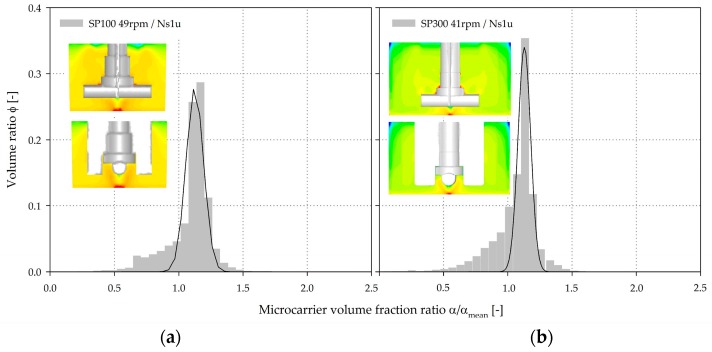
CFD-derived volume-weighted frequency distribution and contour plots of the dimensionless MC volume fraction (*α*/*α_mean_*) at the *Ns1u* (SP100 = 49 rpm (**a**), SP300 = 41 rpm (**b**)).

After determining and validating the fluid flow, the MC distribution was simulated (EE) for a MC solid fraction of 0.1% and for different impeller speeds, in order to compare the two systems. Figure 10 shows an example of the volume-weighted frequency distribution of the dimensionless MC solid fractions (*α*/*α_mean_*) in the two spinner flasks for *Ns1u*. As expected, the highest MC volume fractions (up to 2.8·*α_mean_* for the SP100 and SP300) were, in both cases, found directly below the impeller in the weak mixing zone. This observation was again not surprising because of the definition of *Ns1u*. Even for *Ns1*, a higher MC volume fraction was found in this region, because the MCs must periodically pass through the region in order to swirl up. The spatial position of the CFD-predicted MC deposits agreed well with the observations made during the suspension studies. The CFD-derived volume-weighted frequency distribution of the dimensionless MC volume fractions showed comparable MC homogeneity for the two spinner flask types (*α*/*α_mean_* close to 1). Even though the conditions were comparable in both systems at the vessel bottom for *Ns1u* and *Ns1*, this does not necessarily result in a same MC distribution over the entire vessel volume. The fronting of the distributions clearly indicates zones with low MC volume fractions. These zones were mainly determined near the fluid surface and represent the sedimentation boundary. The similar conditions at the vessel bottom can mainly be explained by the same off-bottom clearance (c = 0.07–0.12), whereas the MC distribution over the entire vessel volume is mostly affected by the d/D ratio. The swirl up of the MCs can be negatively affected by the recirculating fluid flow below the impeller. However, this effect is partially compensated in the two spinner flask types by the low off-bottom clearance. Due to the higher d/D ratio and the slightly different impeller shape of the SP100, recirculating fluid flow were formed near the fluid surface and decreased overall MC homogeneity (see Section 3.2). It seems that this slightly reduced overall MC homogeneity might have an effect on the MC-cell-aggregate formation rate due to the higher probability of particle interactions.

Based on the findings from the EE simulations, additional EL simulations were performed in order to derive the spatial distribution of discrete MC particles and to additionally calculate the circulation time, residence time and the hydrodynamic stresses acting on the particles (see Section 3.5). For this purpose, the two spinner flask types were vertically divided into four zones (∆ h/H_L_ ≈ 0.25) and particle recovery (=percentage of particles per zone) was tracked for each zone. In order to start with “stationary” conditions, the tracking was started after particle movement had been simulated for at least ≥2·$\Theta_{95\%}$. Afterwards, particle recovery in each zone was calculated and averaged over a period of 1·$\Theta_{95\%}$.

**Figure 11:**
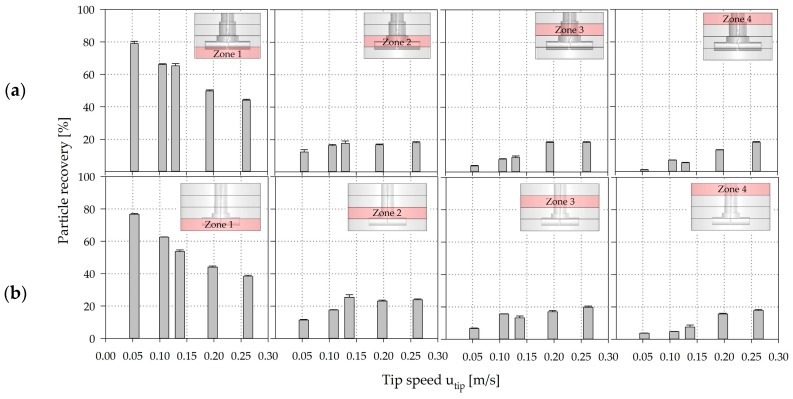
CFD-derived (EL) particle recoveries in the four predefined spinner segments along the axial coordinates (h/H_L_) in the SP100 (**a**) and SP300 (**b**). Zone 1 = 0.00–0.25 h/H_L_, Zone 2 = 0.25–0.50 h/H_L_, Zone 3 = 0.50–0.75 h/H_L_, Zone 4 = 0.75–1.00 h/H_L_.

Figure 11 shows the individual particle recoveries calculated for the four spinner segments. The results revealed that the highest probability of presence of MCs is in the lowest spinner segment. This qualitative observation corresponds well with the results of the suspension studies and the EE simulations. The number of particles in the lower part of the SP100 was 2.8–21.5% higher than in the SP300 for all simulations. However, a clear reduction in the number of particles was observed at higher impeller speeds in both cases, resulting in increased particle recoveries in the other zones (zones 2–4). Interestingly, the particle reduction in zone 1 and the increase in the particle number in zones 2–4 were more significant at lower impeller speeds, showing an asymptotic convergence to absolute homogeneous conditions. Furthermore, the results indicate that the hydrodynamic stresses at the lower part of the spinner flasks in particular have the most significant effect on the cells, because the MCs are most often in this zone. However, the effects of the hydrodynamic stresses in the different zones depended heavily on the particle circulation and residence times (see Section 3.5), demonstrating the dynamics and complexity of the systems.

**Figure 12:**
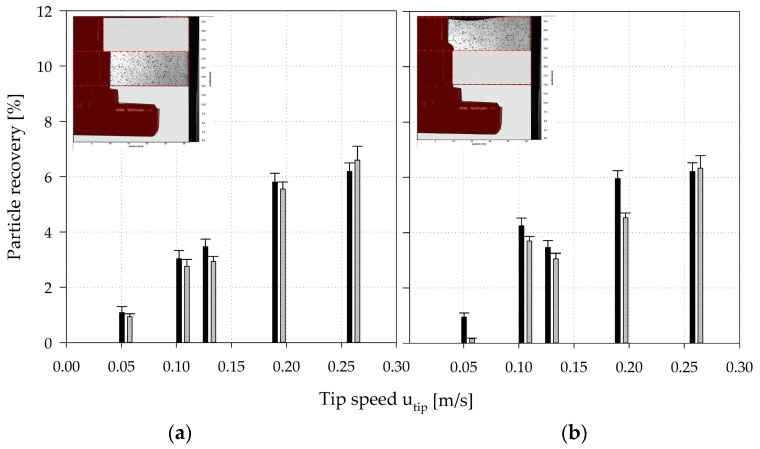
Verification of CFD-predicted particle distributions in zone 3 (**a**) and 4 (**b**) of the SP100. The CFD-predicted particle data was verified by means of shadowgraphy measurements in zones 3 (0.50–0.75 h/H_L_) and 4 (0.75–1.00 h/H_L_). Black bars = CFD data, grey bars = shadowgraphy data.

To verify the applicability of the EL model, the CFD-predicted MC distribution was representatively verified for zones 3 and 4 of the SP100 using shadow imaging measurements (see Figure 12). For this purpose, a discrete number of MCs were added to the spinner flask and the positions of the MCs were captured and subsequently compared with the CFD-derived data. The interrogation space (≈2500–3000 mm^3^) for the particle counting and comparison was defined based on the depth-of-field of the optical lens used for the measurements. The comparison of the modeled and measured particle data in zones 3 and 4 showed good overall agreement, even though the CFD-derived particle values deviated slightly from the measured values (see Figure 12). The observed deviations could be explained by the measurement accuracy of the shadowgraphy method itself or by an overprediction of the turbulence parameters in the transient fluid flow regime in the turbulence models. The reduction in particle recovery in zone 4 from *Ns1u* (*u_tip_* = 0.106 m/s) to *Ns1* (*u_tip_* = 0.130 m/s) can be explained by the fluid flow transition and the generation of a downward oriented recirculating fluid flow structure in the zone. However, the overall agreement was satisfactory and demonstrates the applicability of the EL model for the prediction of particle distribution in the SP100 and SP300. The measured particle velocity components $\vec{u}$ and $\vec{v}$ were also in a comparable range to velocities calculated for particles in the Langarian framework. Thus, no significant differences were found between cell-free and cell-loaded MCs. Therefore, it may be hypothesized that the MCs followed the fluid flow in a predominantly slip-free manner. This statement is also supported by the low sedimentation velocities (1.46 ± 0.56 mm/s) of the MCs.

### 3.5. Circulation Times, Residence Times, and Hydrodynamic Stresses

**Figure 13:**
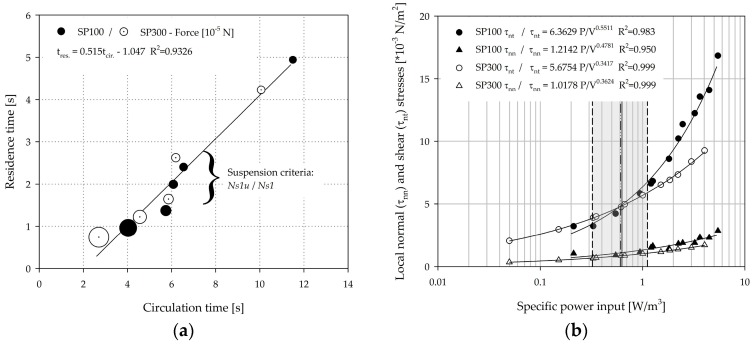
Hydrodynamic stresses. (**a**) Bubble plot showing the relationship between the circulation time, residence time, and the relating particle forces. The size of the bubble indicates the strength of the particle related force. (**b**) Volume-weighted values of local normal and shear stresses in relation to the specific power input. The grey area indicates the range between the *Ns1u* and *Ns1* criteria.

The circulation times and residence times were calculated for each individual spinner segment based on the particle tracking data (from EL simulations) and were subsequently averaged over the four segments (=mean circulation and residence times). Fully sedimented MCs, especially at lower impeller speeds (≤*Ns1u*), were not considered for the analysis. Figure 13a shows the relationship between the mean circulation times and the mean residence times. As expected, the circulation times (2.7–11.5 s) decreased proportionally to the residence times (0.74–4.94 s) as the impeller speed was increased. Interestingly, the proportionality constants for the SP100 = 0.54 and the SP300 = 0.49 were quite similar. This observation can be ascribed to the comparable fluid flow conditions resulting from the comparable geometrical ratios of the two systems. The calculated mean particle forces (see Table 3), which were calculated based on the particle force balance during the simulations, were inversely proportional to the circulation and residence times (see Figure 13a), and are indicated by the size of the circles. This finding is not unexpected, since the specific power input, which was derived from the CFD simulations, increased by approximately the 3rd potency in both spinner flask types. However, an experimental verification of this correlation is difficult due to the low acting torques in the two spinner flasks. Interestingly, the mean values of the particle forces did not change significantly between the lower impeller speeds (<*Ns1u*) and the two suspension criteria, even though the circulation and residence times decreased by up to 50%. Impeller speeds exceeding *Ns1u* and *Ns1* resulted in a slight decrease of the circulation times, although the related particle forces increased logarithmically to the resulting specific power input (see Table 3). The product of the impeller speed and the circulation time resulted in values between 6.0–8.6 (SP100) and 4.5–5.7 (SP300) for impeller speeds exceeding *Ns1u* and *Ns1*. Thus, no constant values were achieved for the investigated conditions. Comparable observations as for the specific power input are also possible when considering the local normal and shear stresses, which were calculated according to Wollny et al. [44] (see Figure 13b). The volume-weighted mean values of the local normal (SP100: 1.15–1.51·10^−3^ N/m^2^, SP300: 0.69–0.88·10^−3^ N/m^2^) and shear stresses (SP100: 4.96–6.62·10^−3^ N/m^2^, SP300: 4.00–4.98·10^−3^ N/m^2^) were in a comparable range in both spinner flask types for impeller speeds between *Ns1u* and *Ns1*. Thus, similar conditions in terms of hydrodynamic stresses can be expected for cultivations in the resulting specific power input range of 0.3–1.1 W/m^3^, which was derived from the simulations. The defined specific power inputs were comparable to the data described by Schirmaier et al. [16], Grein et al. [45], Cierpka et al. [46], and Lawson et al. [17] for benchtop and pilot scale bioreactors, who postulated that specific power inputs of up to 2.1 W/m^3^ are suitable for the MC-based expansion of hMSCs. However, time-dependent hydrodynamic stresses might differ from the two spinner flask types investigated in this study, because the benchtop and pilot scale bioreactors were equipped with axial conveying impellers. It is worth mentioning that the mean values of the local shear stresses in the SP100 increased more than in the SP300 for impeller speeds exceeding *Ns1*. This is mainly caused by the larger d/D ratio, which results in a higher level of turbulence. It is evident that the local shear stresses, which are suspected to cause higher cell damage [47], have a dominant effect over the normal stresses. Moreover, different studies in laminar flow bioreactors have demonstrated that the shear stress can affect the cell morphology and the formation of the extracellular matrix (ECM) [48,49,50]. Yeatts et al. reported that continuous shear stresses of up to 0.15 dynes/cm^2^ (=0.015 Pa) can cause higher expression levels of osteoblastic markers such as osteopontin and osteocalcin [51,52]. However, in most of the cases the hMSCs were exposed to these continuous shear stresses (up to 12 dynes/cm^2^) over a long period (up to 28 days). Thus, the effect of the changing hydrodynamic conditions and the short exposure times in stirred bioreactors needs to be investigated in subsequent studies.

Another popular method for evaluating hydrodynamic stress is based on Kolmogorov’s theory of isotropic turbulence [53,54,55]. While cells in suspension are assumed to only be affected by turbulent eddies of comparable size, those growing on the surface of a MC appear to be more shear sensitive. This might be because they are attached to relatively large particles that are more prone to collisions that might damage the cells. Croughan et al. [56] found that cell damage became significant when the smallest turbulent eddies were approximately two-thirds of the size of a MC. However, to apply Kolmogorov’s theory, the fluid flow must be very turbulent. Taking into account the fact that Re < 10^4^ (see Table 3), the fluid flow is in the transition region of Reynolds numbers, between laminar and fully turbulent conditions. Thus, it would be more reasonable to describe it as moderately turbulent in both cases [31,43]. However, the calculated maximum dissipation rates were higher by a factor of one or two in the impeller swept volume than in the bulk, and therefore, agreed well with findings from the literature [28,57]. As expected, the smallest turbulent eddies were found for the highest tested impeller speeds, with values between 30 µm and 47 µm. In terms of the suspension criteria, the minimum values were predicted in the range of 60 µm and 76 µm, which is much lower than the proposed 2/3 MC size. In contrast, the volume-weighted mean values were slightly higher than the MC size, which demonstrated that only a small proportion of the turbulent eddies are comparable in size to the MCs. This lowers the risk that the MCs might come into contact with these detrimental eddies. However, this fact depends heavily on the resulting circulation times and residence times of the MCs. The mean volume-weighted values for the highest tested impeller speeds were in both cases much closer to the detrimental theoretical value of 141 µm. Even though such eddies occurred at the suspension criteria, the frequency with which the MCs were exposed to such eddies was much lower due to the lower circulation times and residence times (see Section 3.6). Furthermore, in both cases, the volume of *λ* < 141 µm increased from 0.03 to 52.72% and from 0.02 to 63.26% as the impeller speed increased from 25 to 120 rpm and 20 to 100 rpm, respectively.

### 3.6. Cultivation Studies

**Figure 14:**
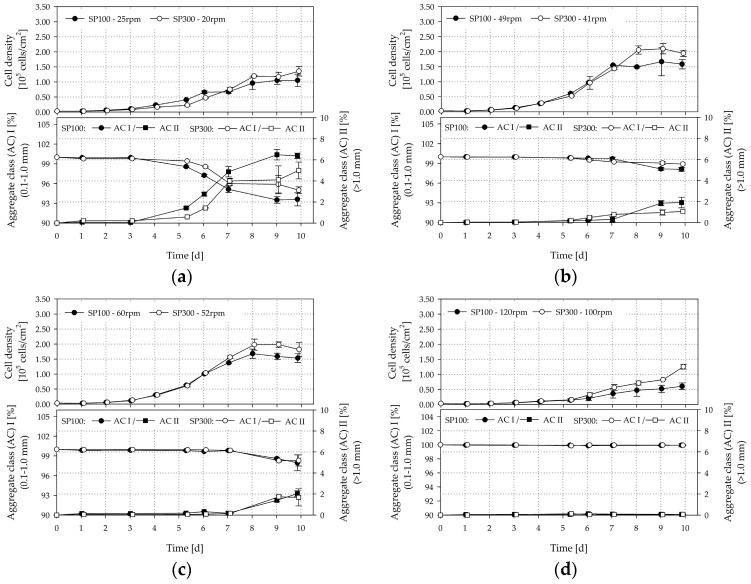
Results of hTERT-ASC cultivations. Time-dependent profiles of living cell densities and MC-cell aggregates. (**a**) SP100 25 rpm/SP300 20 rpm, (**b**) SP100 *Ns1u* 49 rpm/41 rpm, (**c**) SP100 *Ns1* 60 rpm/SP300 52 rpm, (**d**) SP100 120 rpm/SP300 100 rpm; 50% medium exchange on days 4 and 8. Data points were connected for a better overview and do not imply a kinetic relation.

Figure 14a–d shows the time-dependent profiles of living cell densities and MC-cell aggregates for the SP100 and SP300. It can be seen that the investigated hydrodynamic stresses have a significant effect on cell growth and MC-cell-aggregate formation. The highest living cell densities were achieved, of up to 1.68 ± 0.36 × 10^5^ cells/cm^2^ (=6.25 ± 0.35 × 10^5^ cells/mL, EF 56.01) and 2.46 ± 0.16 × 10^5^ cells/cm^2^ (=8.77 ± 0.66 × 10^5^ cells/mL, EF 81.14), in the SP100 and SP300 when working at the suspension criteria. The living peak cell densities in the SP300 were on average up to 40% higher than those in the SP100. Although the two spinner flask types had comparable geometrical ratios, the hydrodynamic stresses in the SP100 were higher at the suspension criteria (see Section 3.5). In fact, the time-dependent stresses were higher due to the lower circulation times, which increases the risk that the cells on the MCs are more frequently exposed to detrimental stresses. At the same time, the residence times, and therefore, the exposure times of the MCs to the time-dependent stresses were shorter. This observation is supported by the slightly lower level of homogeneity in the SP100, as shown in the CFD-simulations. However, in both cases, the peak cell densities were in the same range as cell densities measured in planar, static cultures at maximum confluency (2.9 ± 0.09 × 10^5^ cells/cm^2^, data not shown), in which the cells were expanded in parallel. This result indicates that the cells cultivated at the suspension criteria are mainly restricted by the available growth surface. In contrast, significantly lower cell densities were achieved at lower and higher impeller speeds. A peak living cell density of 1.05 ± 0.06 × 10^5^ cells/cm^2^ (=4.49 ± 0.06 × 10^5^ cells/mL, EF 35.05) and 1.36 ± 0.57 × 10^5^ cells/cm^2^ (=4.88 ± 0.57 × 10^5^ cells/mL, EF 45.20) was determined for the SP100 and the SP300 at 25 rpm and 20 rpm, respectively. These peak cell densities are up to 84% lower than those at the suspension criteria. This observation may be caused by the higher amount of sedimented MCs and the increased MC-cell aggregate formation (see Figure 14a). Although the specific power input for the same tip speed in the SP300 was slightly lower than for the SP100, shorter circulation times and residence times occurred and resulted in reduced MC-cell aggregate formation due to the higher grade of homogeneity. The amount of aggregates with a size of >1.0 mm increased significantly after three days of cultivation at low impeller speeds, which impairs cell growth. We observed that the LDH activity values calculated relative to the values obtained for the suspension criteria on day seven increased by between 32% and 44% in the supernatant. Even, from day 7 to day 10, the LDH activity further increased by up to 60% in all cultivations with impeller speeds between 20 and 60 rpm, which is accompanied by stagnant cell growth after day seven. Comparable results were also found for the cell viability, which was measured for the cells in the supernatant by flow cytometry. The viability of the cells on the MCs was always >99%. This is not surprising as dead cells detach from the MC surface. Thus, the increase of dead cells in the supernatant depends on the cell detachment from the MC surface and the die off of cells in the supernatant. Interestingly, the MC-cell aggregate formation had a stronger influence on the number of dead cells in the supernatant than the hydrodynamic stresses. The percentage of dead cells in the supernatant increased to 58% at the end of the cultivations (day 10) for *N* < *Ns1u*. In contrast, the percentage of dead cells in the supernatant for N > Ns1 was only 30%. This means that day seven represents the optimal point for cell harvesting. In contrast, no significant MC-cell aggregate formation was observed for higher impeller speeds, due to the higher hydrodynamic stress. Although a peak cell density of between 0.6 × 10^5^ cells/cm^2^ and 1.25 × 10^5^ cells/cm^2^ was achieved, the amount of larger aggregates was low, meaning that higher hydrodynamic stresses affected MC-cell aggregation. This was expected and has also been reported by Takahashi et al. [58] and Ferrari et al. [59], who both recommend using impeller speed, among other parameters, to control aggregate size to some extent.

Table 4 shows the main growth-dependent parameters including the cell specific glucose consumption rate, the lactate production rate and the ammonia production rate. By considering the calculated specific glucose consumption rates, it becomes clear that the lowest values were obtained at the suspension criteria in both cases. This is due to the efficient metabolization of glucose under these conditions. The calculated values for the hTERT-ASCs agreed well with those determined by Rafiq et al. [60] for hMSCs in different culture media. The highest specific glucose consumption rates (20.79–35.00 pmol/cell/d) were found at the highest impeller speeds. The relationship between the specific glucose consumption rate and the specific power input can be expressed by a logarithmic function of the 3^rd^ order (*f*(−*q_gluc_*) = −11.085 + [−1.311 × *ln*(*P*/*V*)] + [−3.529 × *ln*(*P*/*V*)]^2^ + [−0.806 × *ln*(*P*/*V*)]^3^, *R*^2^ = 0.963), whereas this correlation is only valid for the investigated P/V range. Similar correlations were also calculated for the cell specific lactate and ammonia production rates, where values of up to 193% and 170% higher than those in the spinner flasks at the suspension criteria were determined at the highest impeller speeds. These higher values indicate that the cells are more stressed at higher impeller speeds as a result of higher hydrodynamic loads. Moreover, such high metabolite production rates also result in a large accumulation of inhibitory metabolites during cultivation and may reduce cell yield [39,61,62]. The different obtained correlations were used as initial parameters for the growth modelling (see Section 3.6).

**Figure 15:**
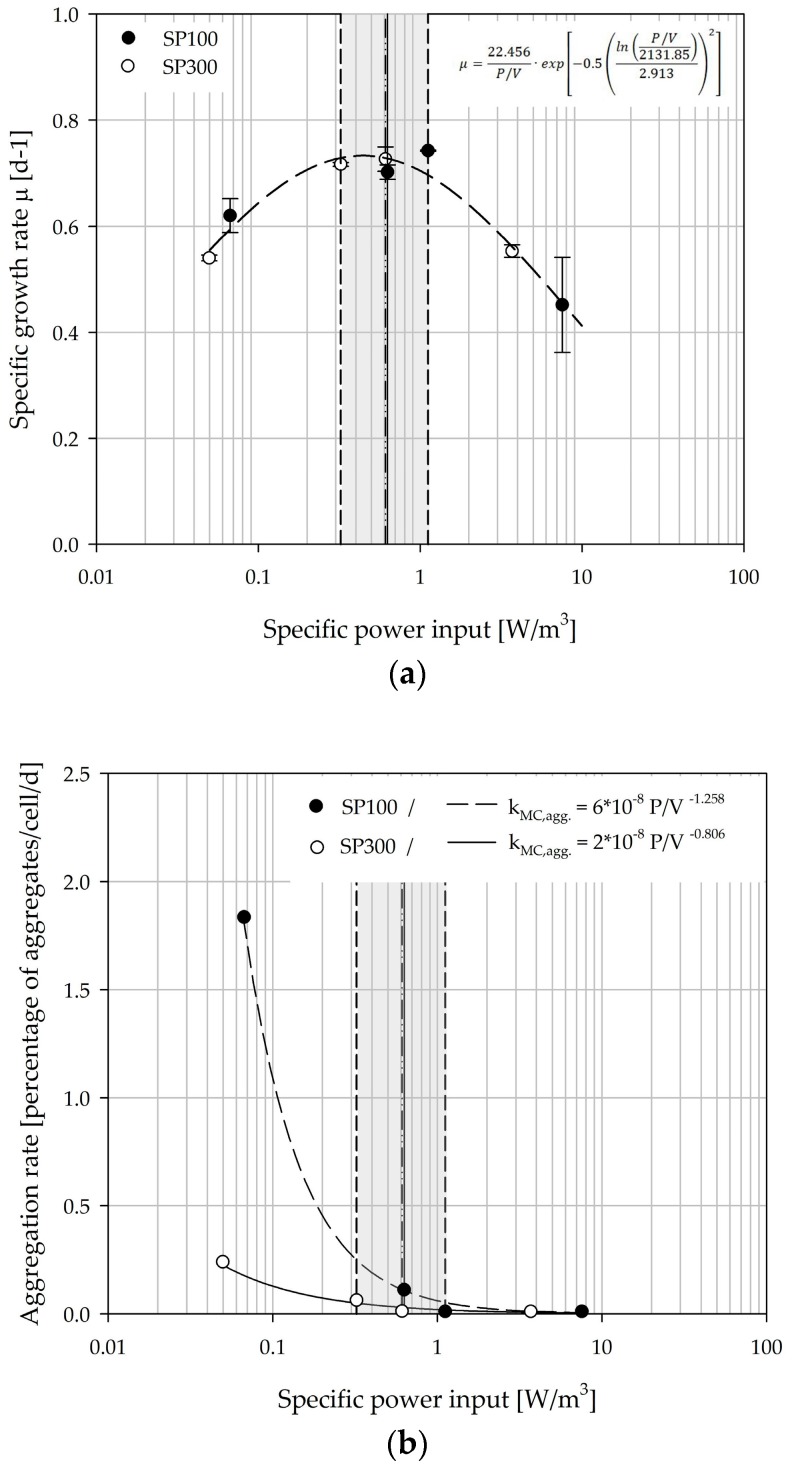
Dependency of the specific growth rate (**a**) and the MC-cell aggregation rate (**b**) on the specific volumetric power input. The grey marked area indicates the range between *Ns1u* and *Ns1*.

Figure 15a shows the relationship between the overall mean specific growth rate and the specific power input. The parabolic curve profile of the specific growth rate shows optimal cell growth between *Ns1u* and *Ns1*. For specific power inputs between 0.33 and 1.12 W/m^3^, maximum values for µ between 0.70 and 0.74 d^−1^ (=0.93–0.99 d) were achieved. Comparable growth rates for hTERT-MSCs were also described by Balducci et al. [63], Leber et al. [64], and Cierpka et al. [46]. Moreover, the maximum specific growth rates correlate quite well with the values (0.70 ± 0.02 d^−1^) from experiments in the planar and static culture systems (data not shown). This demonstrates the comparability of the two spinner flask types, even though slight deviations exist. Similar relationships to those for the specific growth rate and the specific power input were also found for other hydrodynamic stress parameters (i.e., *l_λ_, LSS, F*; see Table 3). The derived correlations represent the basis for growth modeling and future scale-up investigations.

Based on the measured MC-cell aggregates, a cell specific aggregation rate was derived from the data. For this purpose, the data of the class II aggregate (>1.0 mm) was correlated with cell growth over the time. The dependency of the MC-cell aggregation rate on specific power input is shown in Figure 15b. The determined MC-cell aggregation rates for the SP100 were higher than for the SP300 for all investigated conditions. However, this was not surprising because of the lower MC homogeneity, especially at lower impeller speeds (*N* < *Ns1u* and *Ns1*).

Table 5 shows the results of the flow cytometric measurements after cell harvesting for the four negative (CD14^−^, CD20^−^, CD34^−^, and CD45^−^) and the three positive (CD73^+^, CD90^+^, and CD105^+^) markers. In order to visualize a change in the marker expression profile, the results were compared with those obtained from the cell inoculum. All positive markers were strongly expressed (>91%) and the negative markers exhibited a lack of expression (<2.7%). These results correspond with measurements for hTERT-ASCs by Balducci et al. [63], Yin et al. [65], and Wolbank et al. [66]. Moreover, the results are in good agreement with the minimal expression levels for hASCs recommended by the International Society of Cellular Therapy (ISCT) and the International Federation for Adipose Therapeutics and Science (IFATS) [67]. Statistically significant differences were found in all spinner flask cultivations for CD73, CD90, and CD105, when they were compared with the expression levels obtained from the cell inoculum. However, a correlation of the surface marker expression levels with the hydrodynamic stress or MC-cell aggregate formation was not found.

### 3.7. Growth Modelling

**Figure 16:**
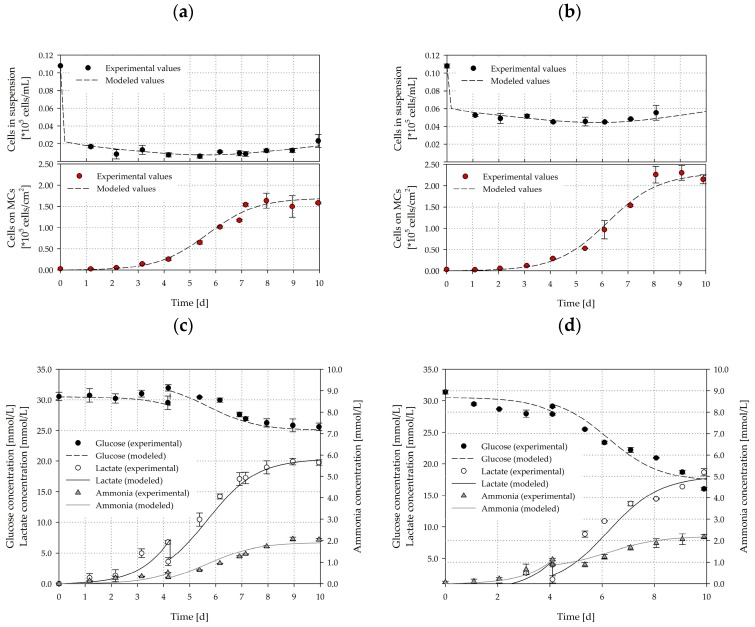
Comparison of experimental and simulated data for cell density (**a**,**b**), substrate, and metabolites (**c**,**d**). The growth simulations were performed for cultivations (*n* = 3) in the SP100 (left) and SP300 (right) at the *Ns1u* criterion. The symbols represent the experimentally measured values collected by offline measurements. The lines represent the simulated time course.

To test the validity of the unstructured, segregated, simplistic growth model for the SP100 and SP300, independent cultivation runs (*n* = 3 per spinner flask type) were performed at the *Ns1u* criterion. To simulate the cell density, the substrate and the metabolites, the parameters determined in the previous cultivations were used (see Section 3.5). Figure 16 shows the measured values and the simulated time courses for the cell density (a–b), the substrate and the metabolites (c–d). The simulated time courses show good overall agreement with the experimentally measured values and demonstrate the applicability of the unstructured, segregated growth model. By using the determined growth parameters from the cultivation study, the cell growth, glucose consumption, lactate production, and ammonia production could be well approximated. The greatest deviations in cell density were in the range of 3–20% for the cells in suspension and 4–24% for the cells on the MCs. However, the accuracy of the measured cell densities decreased towards the end of the cultivations due to the formation of larger aggregates (see Section 3.5). The intensified aggregate formation and its effect on the growth surface was not considered in the model. However, the principal growth behaviors were well captured. Moreover, the cell densities measured in both systems were comparable to the previous cultivations (see Section 3.5), and therefore, demonstrated the reproducibility of the cultivation processes, especially when using the stable hTERT-ASCs cell line. Furthermore, the glucose, lactate and ammonia time courses, agreed well, even though the determined specific substrate consumption and metabolite production rates were prone to errors due to the accuracy of the measurement method. The lactate and ammonia time courses show that the growth associated assumption was valid and can be used for modelling because the production of these two substances was directly linked to the cell number increase.

## 4. Conclusions

In this study, extensive numerical and experimental investigations were carried out for two spinner flask types with comparable geometrical ratios. For this purpose, comprehensive multi-phase CFD simulations based on EE and EL models were performed and the derived hydrodynamic stress parameters were linked with growth-related parameters to create an unstructured, segregated, simplistic growth model. Providing the numerical models are valid, CFD modeling is one of the most effective techniques for characterization of flow fields. However, multi-phase CFD simulations, which take the MCs into account, have not been used very often for the prediction of MC-related stress parameters. Most of the previous CFD-based investigations only focused on single-phase flows, which might be driven by the higher computational requirements for multi-phase simulations and their economical relevance. However, it is widely accepted that hMSCs are exposed to different hydrodynamic stress levels during their lifespan in a stirred bioreactor. Thus, particle-related data, like the MC circulation and residence times in high shear zones, among others, are meaningful and will become more important in the future. They allow the bioreactor design to be improved and the optimum growth parameters to yield relevant amounts of therapeutic cells to be determined.

In order to test the influence of different hydrodynamic stress levels on cell growth and cell quality, CFD simulations were performed for different impeller speeds and for the suspension criteria (*Ns1u*, *Ns1*), which were determined experimentally. The suspension studies clearly showed the linear relationship between MC concentration and the tip speed required for *Ns1u*/*Ns1*. The resulting correlation from the multi-regression analysis can be used for further studies with Corning^®^ spinner flasks using either the same or other polystyrene-based MCs (with comparable properties). Due to the comparable geometrical ratios of the two spinner flask types used in this study, suspension criteria were fulfilled at the same tip speed. However, the CFD simulations indicated that this does not necessarily result in the same overall MC homogeneity in the two systems, because the criteria only consider conditions at the bottom of the reactor. The slightly reduced overall MC homogeneity at *Ns1u* and *Ns1* in the SP100 not only affects cell growth but also aggregate formation to some extent, even though the two systems had comparable geometrical dimensions. The observed deviations in cell growth and aggregate formation were mainly due to slight differences in the d/D and c/D ratios. Hence, the investigations demonstrated the dependency of the suspension criteria on the geometrical dimensions, findings that can serve as a basis for further scale-up studies. Nevertheless, optimal cultivation conditions were found for *Ns1u* < *N* < *Ns1*, which corresponded to specific power inputs between 0.3 and 1.1 W/m^3^. Although, all numerical methods, including CFD, use mathematical assumptions and empirical variables, the experimental PIV and shadowgraphy measurements demonstrated their accuracy and usefulness. In all cases, there was sufficient and satisfactory comparability between the experimental and the modeled data, underlining the meaningfulness of the derived hydrodynamic parameters (e.g., *P*/*V*, *τ_nt_*, *F_MC_*, etc.). Based on these parameters and their correlation with the growth-related data, stress dependent correlations (e.g., µ vs. *P*/*V* or *τ_nt_*, −*q_i_* vs. *P*/*V* or *τ_nt_*, etc.) were, for the first time, derived for hTERT-ASCs. The correlations were used to set-up an unstructured, segregated growth model with very well matching time courses compared to the experimental data for cell growth, glucose consumption, lactate production and ammonia production. Maximum deviations between the simulated and measured cell densities for cells growing on the MC surface were in the range of 4 to 24%. This means that the descriptiveness and predictive power of the model is satisfactory, especially when considering the accuracy of the experimentally measured values. The intensified aggregate formation was not considered in the growth model. Nevertheless, good agreement has already been achieved. Subsequent investigations are necessary to understand the aggregation dynamics under different conditions in more detail and to consider their effects in the growth model. In addition, the applicability of the derived stress-dependent correlations for hTERT-ASCs in other systems and their transferability to primary hMSCs should be studied in subsequent work. For primary hMSCs, physiological states such as the replicative senescence should be considered in the growth model. This might be done by incorporating a senescence-related inhibition factor or by using population modelling approaches (e.g., proliferating population vs. senescent cell population). However, further investigations are necessary to determine such senescence-related factors, which can then be incorporated in a mathematical model.

## Figures and Tables

**Table 1:** Main physical dimensions and ratios of the two Corning^®^ spinner flask types.

Physical Dimension/Ratios		Corning^®^ 125 mL Spinner	Corning^®^ 500 mL Spinner
D *	[mm]	63.5	87.3
H_L_ **	[mm]	41	52
H *	[mm]	145	203
V_max_ *	[mL]	100	300
d_max_ *	[mm]	41.5	50.3
C **	[mm]	8.0	8.0
β	[°]	90	90
H/D	[-]	0.65	0.60
d/D	[-]	0.68	0.58
c/D	[-]	0.12	0.07

* Values from manufacturer description. ** Measured values from computer-aided design model.

**Table 2:** Parameters used for growth modelling.

Parameter	Values	Reference
−*q_Glc_* (pmol/cell/d)	9.8–35	This study
*q_Lac_* (pmol/cell/d)	20–89	This study
*q_Amn_* (pmol/cell/d)	6–19	This study
*k_at_* (d^−1^)	0.033–0.05	This study
*k_det_* (d^−1^)	0.002–0.01	This study
*K_Glc_* (mmol/L)	0.4	[37,39]
*K_Lac_* (mmol/L)	35–50	[37,39]
*K_Amn_* (mmol/L)	8–10	[37,39]

**Table 3:** Overview of the main biochemical engineering parameters for the SP100 and SP300. Parameters were obtained from the CFD simulations.

*N*	*u_tip_*	*Re*	*P/V*	*l_λ_^(a)^*	*LSS ^(b)^*	*LNS ^(c)^*	*F ^(d)^*
(rpm)	(m/s)	(-)	(W/m^3^)	(μm)	(10^−3^ N/m^2^)	(10^−3^ N/m^2^)	(10^−5^ N)
**Corning^®^ 125 mL spinner (SP100):**							
25	0.054	715	0.07	130/530	3.21/69.41	1.04/43.17	0.75
49	0.106	1402	0.63	66/228	4.96/187.00	1.15/109.00	0.85
60	0.130	1717	1.12	60/191	6.62/232.37	1.51/127.20	0.91
120	0.261	3434	7.56	30/111	13.55/437.69	2.33/277.44	1.82
**Corning^®^ 500 mL spinner (SP300):**							
20	0.053	841	0.05	136/546	2.05/214.40	0.35/138.86	0.83
41	0.108	1724	0.33	76/295	4.00/481.99	0.69/362.76	0.89
52	0.137	2186	0.61	66/282	4.98/680.55	0.88/473.87	1.04
100	0.263	4204	3.70	47/181	9.28/1352.86	1.71/874.34	2.10

*^(a)^* Volume-weighted minimum/mean values of turbulent Kolmogorov length scale. *^(b,c)^* Local shear (LSS) and normal (LNS) stress for volume-weighted mean/maximum values. *^(d)^* Mean values of the force acting on particles weighted by number.

**Table 4:** Overview of the main growth-dependent parameters in the SP100 and SP300.

N	Living *x_max_*	EF	µ/*t_d_*	−*q_gluc_*	*q_lac_*	*q_NH4_^+^*
(rpm)	(10^5^ Cells/mL) (10^5^ cells/cm^2^)	(-)	(d^−1^) (d)	(pmol cell^−1^ d^−1^)		
**Corning^®^ 125 mL Spinner (SP100):**						
25	4.49 ± 0.06 1.05 ± 0.06	35.03	0.62 ± 0.03 1.12 ± 0.06	−13.21 ± 2.27	20.65 ± 2.73	8.78 ± 0.28
49	6.01 ± 0.12 1.67 ± 0.12	55.62	0.70 ± 0.01 0.99 ± 0.02	−10.55 ± 1.59	35.22 ± 1.91	6.09 ± 0.42
60	6.25 ± 0.35 1.68 ± 0.36	56.01	0.74 ± 0.01 0.93 ± 0.01	−9.80 ± 0.76	30.28 ± 1.01	6.20 ± 0.34
120	2.17 ± 0.40 0.60 ± 0.04	20.11	0.45 ± 0.09 1.53 ± 0.38	−35.00 ± 1.61	88.78 ± 5.21	16.48 ± 0.25
**Corning^®^ 500 mL spinner (SP300):**						
20	4.88 ± 0.57 1.36 ± 0.57	45.20	0.54 ± 0.01 1.28 ± 0.01	−20.98 ± 0.93	28.60 ± 9.86	14.71 ± 0.15
41	8.51 ± 0.16 2.46 ± 0.16	81.92	0.72 ± 0.01 0.97 ± 0.01	−15.47 ± 0.59	40.63 ± 1.78	10.64 ± 0.54
52	8.77 ± 0.66 2.43 ± 0.66	81.14	0.73 ± 0.02 0.95 ± 0.03	−11.75 ± 1.23	35.29 ± 3.28	9.73 ± 0.42
100	4.51 ± 0.29 1.25 ± 0.29	41.76	0.55 ± 0.01 1.25 ± 0.03	−20.76 ± 9.84	88.56 ± 2.09	18.96 ± 1.39

**Table 5:** Results of flow cytometric measurements. The results show the percentage of positive cells for a given surface marker. A one-way ANOVA (Holm–Sidak method, *n* = 3; *p* < 0.05) with multiple comparisons versus the control group was performed for the statistical analysis.

Marker	Inoculum	SP100				SP300			
	T-flasks	25 rpm	49 rpm	60 rpm	120 rpm	20 rpm	41 rpm	52 rpm	100 rpm
	(%)	(%)				(%)			
**CD14^−^**	2.7	1.7 ± 0.2	1.3 ± 0.2	1.0 ± 0.3	1.2 ± 0.0	1.9 ± 0.3	1.5 ± 0.3	1.3 ± 0.1	1.2 ± 0.1
		(*p* 0.518)	(*p* 0.100)	(*p* 0.442)	(*p* 0.493)	(*p* > 0.05)	(*p* > 0.05)	(*p* > 0.05)	(*p* > 0.05)
**CD20^−^**	2.7	1.7 ± 0.2	1.3 ± 0.2	1.0 ± 0.3	1.2 ± 0.0	1.9 ± 0.3	1.5 ± 0.3	1.3 ± 0.1	1.2 ± 0.1
		(*p* 0.518)	(*p* 0.100)	(*p* 0.442)	(*p* 0.493)	(*p* > 0.05)	(*p* > 0.05)	(*p* > 0.05)	(*p* > 0.05)
**CD34^−^**	2.7	1.7 ± 0.2	1.3 ± 0.2	1.0 ± 0.3	1.2 ± 0.0	1.9 ± 0.3	1.5 ± 0.3	1.3 ± 0.1	1.2 ± 0.1
		(*p* 0.518)	(*p* 0.100)	(*p* 0.442)	(*p* 0.493)	(*p* > 0.05)	(*p* > 0.05)	(*p* > 0.05)	(*p* > 0.05)
**CD45^−^**	2.7	1.7 ± 0.2	1.3 ± 0.2	1.0 ± 0.3	1.2 ± 0.0	1.9 ± 0.3	1.5 ± 0.3	1.3 ± 0.1	1.2 ± 0.1
		(*p* 0.518)	(*p* 0.100)	(*p* 0.442)	(*p* 0.493)	(*p* > 0.05)	(*p* > 0.05)	(*p* > 0.05)	(*p* > 0.05)
**CD73^+^**	99.4	99.6 ± 0.0	99.8 ± 0.0	97.1 ± 2.8	99.6 ± 0.2	99.5 ± 0.1	99.7 ± 0.0	99.8 ± 0.1	99.7 ± 0.0
		(*p* < 0.05)	(*p* < 0.05)	(*p* < 0.05)	(*p* < 0.05)	(*p* < 0.05)	(*p* < 0.05)	(*p* < 0.05)	(*p* < 0.05)
**CD90^+^**	96.2	97.7 ± 0.4	98.9± 0.0	98.7 ± 0.2	97.6 ± 0.7	95.0 ± 0.0	96.1 ± 0.8	97.5 ± 0.4	96.8 ± 0.1
		(*p* < 0.05)	(*p* < 0.05)	(*p* < 0.05)	(*p* < 0.05)	(*p* < 0.05)	(*p* < 0.05)	(*p* < 0.05)	(*p* < 0.05)
**CD105^+^**	99.3	91.7 ± 1.3	94.6 ± 0.4	94.0 ± 0.8	96.4 ± 2.1	94.0 ± 0.5	91.4 ± 2.4	97.6 ± 0.2	98.7 ± 0.4
		(*p* < 0.05)	(*p* < 0.05)	(*p* < 0.05)	(*p* < 0.05)	(*p* < 0.05)	(*p* < 0.05)	(*p* < 0.05)	(*p* < 0.05)

## Latin Symbols

*Amn*	(mmol/L)	Ammonia concentration
*A_p_*	(cm^2^/g)	Specific surface per unit mass MC
*c*	(m)	Off-bottom clearance
*c_MC_*	(g/L)	Microcarrier concentration
*C_D_*	(-)	Drag coefficient
*d*	(m)	Impeller diameter
*d_P_*	(m)	Particle diameter
*D*	(m)	Vessel diameter
*EF*	(-)	Expansion factor
$\vec{F}$	(N)	Force (vector)
${\vec{F}}_{D}$	(N)	Drag force (vector)
*g*	(m/s^2^)	Gravitational acceleration
*Glc*	(mmol/L)	Glucose concentration
*H*	(m)	Vessel height
*H_L_*	(m)	Liquid height
*k_at_*	(d^−1^)	Cell attachment constant
*k_det_*	(d^−1^)	Cell detachment constant
*K_Amn_*	(mmol/L)	Inhibition constant of ammonia
*K_Glc_*	(mmol/L)	Monod constant of glucose
*K_Lac_*	(mmol/L)	Inhibition constant of lactate
*Lac*	(mmol/L)	Lactate concentration
*m_Glc_*	(mmol/cell/d)	Glucose consumption rate for maintenance
*Ns1u, Ns1*	(rpm)	Suspension criteria
*p*	(Pa)	Pressure
*PDL*	(-)	Population doubling level
*q_Amn_*	(mmol/cell/d)	Specific ammonia production rate
*−q_Glc_*	(mmol/cell/d)	Specific glucose consumption rate
*q_Lac_*	(mmol/cell/d)	Specific lactate production rate
*Re_p_*	(-)	Reynolds number for a particle
$\vec{u}$	(m/s)	Velocity (vector)
*u_tip_*	(m/s)	Tip speed
${\vec{u}}_{r,s}$	(-)	Terminal velocity correlation for the solid phase (acc. to Symlal and O’Brien)
*V_max_*	(m^3^)	Maximum working volume
*X_MC_*	(cells/cm^2^)	Cells on microcarrier
*X_max_*	(cm^2^)	Maximum growth surface
*X_Sus_*	(cells/mL)	Cell in suspension
*X_V_*	(cells/mL)	Viable cells
*x, y, z*	(m)	Spatial co-ordinates
*Y_x/Glc_*	(cells/mmol)	Cell specific yield factor of glucose
Greek symbols		
*α*	(-)	Phase volume fraction
*α_mean_*	(-)	Mean phase volume fraction
*β*	(°)	Impeller blade angle
*μ*	(d^−1^)	Specific growth rate
*μ_max_*	(d^−1^)	Maximum specific growth rate
*ρ*	(kg/m^3^)	Density
*σ_(Glc)_*	(-)	Simulation step response
$\bar{\tau }$	(N/m^2^)	Reynolds stress tensor
$\tau_{nt}$	(N/m^2^)	Local shear stress
∇	(-)	Nabla operator

## References

[1] Heathman T.R.J., Nienow A.W., McCall M.J., Coopman K., Kara B., Hewitt C.J. (2015). The translation of cell-based therapies: Clinical landscape and manufacturing challenges. Regen. Med..

[2] Ratcliffe E., Glen K.E., Naing M.W., Williams D.J. (2013). Current status and perspectives on stem cell-based therapies undergoing clinical trials for regenerative medicine: Case studies. Br. Med. Bull..

[3] Weiss M.L., Rao M.S., Deans R., Czermak P. (2016). Manufacturing Cells for Clinical Use. Stem Cells Int..

[4] Trounson A., McDonald C. (2015). Stem Cell Therapies in Clinical Trials: Progress and Challenges. Cell Stem Cell.

[5] Wei X., Yang X., Han Z., Qu F., Shao L., Shi Y. (2013). Mesenchymal stem cells: A new trend for cell therapy. Acta Pharmacol. Sin..

[6] Abdallah B.M., Kassem M. (2008). Human mesenchymal stem cells: From basic biology to clinical applications. Gene Ther..

[7] Malik N.N., Durdy M.B. (2015). Cell Therapy Landscape. Translational Regenerative Medicine.

[8] Simaria A.S., Hassan S., Varadaraju H., Rowley J., Warren K., Vanek P., Farid S.S. (2014). Allogeneic cell therapy bioprocess economics and optimization: Single-use cell expansion technologies. Biotechnol. Bioeng..

[9] Jossen V., van den Bos C., Eibl R., Eibl D. (2018). Manufacturing human mesenchymal stem cells at clinical scale: Process and regulatory challenges. Appl. Microbiol. Biotechnol..

[10] Lodge A., Detela G., Barry J., Ginty P., Mount N. (2017). Global Regulatory Perspective for MSCs. Mesenchymal Stem Cells.

[11] Eibes G., dos Santos F., Andrade P.Z., Boura J.S., Abecasis M.M., da Silva C.L., Cabral J.M. (2010). Maximizing the ex vivo expansion of human mesenchymal stem cells using a microcarrier-based stirred culture system. J. Biotechnol..

[12] Lipsitz Y.Y., Milligan W.D., Fitzpatrick I., Stalmeijer E., Farid S.S., Tan K.Y., Smith D., Perry R., Carmen J., Chen A. (2017). A roadmap for cost-of-goods planning to guide economic production of cell therapy products. Cytotherapy.

[13] Sharma S., Raju R., Shiu S. (2011). Stem cell culture engineering—Process scale-up and beyond. Biotechnol. J..

[14] Kino-Oka M., Mizutani M. (2016). Cell Production System Based on Flexible Modular Platform. Stem Cell Manufacturing.

[15] Badenes S.M., Fernandes-Platzgummer A., Rodrigues C.A.V., Diogo M.M., da Silva C.L., Cabral J.M.S. (2016). Microcarrier Culture Systems for Stem Cell Manufacturing. Stem Cell Manufacturing.

[16] Schirmaier C., Jossen V., Kaiser S.C., Jüngerkes F., Brill S., Safavi-Nab A., Siehoff A., van den Bos C., Eibl D., Eibl R. (2014). Scale-up of adipose tissue-derived mesenchymal stem cell production in stirred single-use bioreactors under low-serum conditions. Eng. Life Sci..

[17] Lawson T., Kehoe D.E., Schnitzler A.C., Rapiejko P.J., Der K.A., Philbrick K., Punreddy S., Rigby S., Smith R., Feng Q. (2017). Process development for expansion of human mesenchymal stromal cells in a 50L single-use stirred tank bioreactor. Biochem. Eng. J..

[18] Abraham E., Gupta S., Jung S., McAfee E. (2017). Bioreactor for Scale-Up: Process Control. Mesenchymal Stem Cells.

[19] Werner S., Kaiser S.C., Kraume M., Eibl D. (2014). Computational fluid dynamics as a modern tool for engineering characterization of bioreactors. Pharm. Bioprocess.

[20] Kaiser S., Löffelholz C., Werner S., Eibl D., Minin I.V., Minin O.V. (2011). CFD for characterizing standard and single-use stirred cell culture bioreactors. Computational Fluid Dynamics.

[21] Eibl R., Kaiser S., Lombriser R., Eibl D. (2010). Disposable bioreactors: The current state-of-the-art and recommended applications in biotechnology. Appl. Microbiol. Biotechnol..

[22] Sucosky P., Osorio D.F., Brown J.B., Neitzel G.P. (2004). Fluid mechanics of a spinner-flask bioreactor. Biotechnol. Bioeng..

[23] Nienow A.W., Rielly C.D., Brosnan K., Bargh N., Lee K., Coopman K., Hewitt C.J. (2013). The physical characterisation of a microscale parallel bioreactor platform with an industrial CHO cell line expressing an IgG4. Biochem. Eng. J..

[24] Sharma C., Malhotra D., Rathore A.S. (2011). Review of Computational fluid dynamics applications in biotechnology processes. Biotechnol. Prog..

[25] Kaiser S., Jossen V., Schirmaier C., Eibl D., Brill S., van den Bos C., Eibl R. (2013). Fluid Flow and Cell Proliferation of Mesenchymal Adipose-Derived Stem Cells in Small-Scale, Stirred, Single-Use Bioreactors. Chem. Ing. Tech..

[26] Jossen V., Kaiser S.C., Schirmaier C., Herrmann J., Tappe A., Eibl D., Siehoff A., den Bos C.V., Eibl R. (2014). Modification and qualification of a stirred single-use bioreactor for the improved expansion of human mesenchymal stem cells at benchtop scale. Pharm. Bioprocess.

[27] Jossen V., Schirmer C., Mostafa Sindi D., Eibl R., Kraume M., Pörtner R., Eibl D. (2016). Theoretical and Practical Issues That Are Relevant When Scaling Up hMSC Microcarrier Production Processes. Stem Cells Int..

[28] Liovic P., Šutalo I.D., Stewart R., Glattauer V., Meagher L. Fluid flow and stresses on microcarriers in spinner flask bioreactors. Proceedings of the Ninth International Conference on CFD in the Minerals and Process Industries.

[29] Ismadi M.-Z., Hourigan K., Fouras A. (2014). Experimental Characterisation of Fluid Mechanics in a Spinner Flask Bioreactor. Processes.

[30] Hutmacher D.W., Singh H. (2008). Computational fluid dynamics for improved bioreactor design and 3D culture. Trends Biotechnol..

[31] Berry J.D., Liovic P., Šutalo I.D., Stewart R.L., Glattauer V., Meagher L. (2016). Characterisation of stresses on microcarriers in a stirred bioreactor. Appl. Math. Model..

[32] Liovic P., Šutalo I.D., Meagher L., Lovrecz G.O. Computations of flow environments in medium-scale stirred tank bioreactors for stem cell expansion. Proceedings of the 2014 12th International Conference on Nanochannels, Microchannels, and Minichannels.

[33] Julaey M., Hosseini M., Amani H. (2016). Stem Cells Culture Bioreactor Fluid Flow, Shear Stress and Microcarriers Dispersion Analysis Using Computational Fluid Dynamics. J. Appl. Biotechnol. Rep..

[34] Symlal M., Rogers W., O’Brien T.J. (1989). Computer Simulation of Bubbles in a Fluidized Bed. AIChE Symp..

[35] Schiller L., Naumann Z. (1935). A drag coefficient correlation. Z. Ver. Deutsch. Ing..

[36] (2010). Ansys Fluent 13.0. Theory Guide.

[37] Möhler L., Bock A., Reichl U. (2008). Segregated mathematical model for growth of anchorage-dependent MDCK cells in microcarrier culture. Biotechnol. Prog..

[38] Bock A., Sann H., Schulze-Horsel J., Genzel Y., Reichl U., Möhler L. (2009). Growth behavior of number distributed adherent MDCK cells for optimization in microcarrier cultures. Biotechnol. Prog..

[39] Schop D., Janssen F.W., van Rijn L.D., Fernandes H., Bloem R.M., de Bruijn J.D., van Dijkhuizen-Radersma R. (2009). Growth, metabolism, and growth inhibitors of mesenchymal stem cells. Tissue Eng. Part A.

[40] Zwietering T.N. (1958). Suspending solid particles in liquid by agitators. Chem. Eng. Sci..

[41] Liepe F., Sperling R., Jembere S. (1998). Rührwerke: Theoretische Grundlagen, Auslegung und Bewertung.

[42] LaVision (2015). ParticleMaster Shadow: Product-Manual.

[43] Venkat R.V., Stock L.R., Chalmers J.J. (1996). Study of hydrodynamics in microcarrier culture spinner vessels: A particle tracking velocimetry approach. Biotechnol. Bioeng..

[44] Wollny S. (2010). Experimentelle und Numerische Untersuchungen zur Partikelbeanspruchung in Gerührten (Bio-) Reaktoren. Ph.D. Thesis.

[45] Grein T.A., Leber J., Blumenstock M., Petry F., Weidner T., Salzig D. (2016). Multiphase mixing characteristics in a microcarrier-based stirred tank bioreactor suitable for human mesenchymal stem cell expansion. Process Biochem..

[46] Cierpka K., Elseberg C.L., Niss K., Kassem M., Salzig D., Czermak P. (2013). hMSC Production in Disposable Bioreactors with Regards to GMP and PAT. Chem. Ing. Tech..

[47] Langer G., Deppe A. (2000). Zum Veständnis der hydrodynamischen Beanspruchung von Partikeln in turbulenten Rührerströmungen. Chem. Ing. Tech..

[48] Weyand B., Reimers K., Vogt P.M. Influences of Extracellular Matrix Properties and Flow Shear Stresses on Stem Cell Shape in a Three-Dimensional Dynamic Environment. Proceedings of the 8th International Conference on Cell & Stem Cell Engineering (ICCE).

[49] Weyand B., Israelowitz M., von Schroeder H.P., Vogt P.M. (2009). Fluid Dynamics in Bioreactor Design: Considerations for the Theoretical and Practical Approach. Adv. Biochem. Eng. Biotechnol..

[50] Weyand B., Kasper C., Israelowitz M., Gille C., von Schroeder H.P., Reimers K., Vogt P.M. (2012). A Differential Pressure Laminar Flow Reactor Supports Osteogenic Differentiation and Extracellular Matrix Formation from Adipose Mesenchymal Stem Cells in a Macroporous Ceramic Scaffold. Biores. Open Access.

[51] Yeatts A.B., Choquette D.T., Fisher J.P. (2013). Bioreactors to influence stem cell fate: Augmentation of mesenchymal stem cell signaling pathways via dynamic culture systems. Biochim. Biophys. Acta Gen. Subj..

[52] Yeatts A.B., Fisher J.P. (2011). Bone tissue engineering bioreactors: Dynamic culture and the influence of shear stress. Bone.

[53] Nienow A. (2010). Scale-Up, Stirred Tank Reactors. Encyclopedia of Industrial Biotechnology: Bioprocess, Bioseparation, and Cell Technology.

[54] Nienow A.W. (2006). Reactor engineering in large scale animal cell culture. Cytotechnology.

[55] Thomas C.R., Zhang Z., Galindo E., Ramirez O.T. (1998). The Effect of Hydrodynamics on Biological Materials. Advances in Bioprocess Engineering.

[56] Croughan M.S., Hamel J.-F., Wang D.I.C. (2006). Hydrodynamic effects on animal cells grown in microcarrier cultures. Biotechnol. Bioeng..

[57] Ponnuru K., Wu J., Ashok P., Tzanakakis E., Furlani E.P. (2014). Analysis of Stem Cell Culture Performance in a Microcarrier Bioreactor System. Proc. Int. NSTI Nanotech. Conf..

[58] Takahashi I., Sato K., Mera H., Wakitani S., Takagi M. (2016). Effects of agitation rate on aggregation during beads-to-beads subcultivation of microcarrier culture of human mesenchymal stem cells. Cytotechnology.

[59] Ferrari C., Balandras F., Guedon E., Olmos E., Chevalot I., Marc A. (2012). Limiting cell aggregation during mesenchymal stem cell expansion on microcarriers. Biotechnol. Prog..

[60] Rafiq Q.A., Ruck S., Hanga M.P., Heathman T.R., Coopman K., Nienow A.W., Williams D.J., Hewitt C.J. (2017). Qualiative and quantitative demonstration of bead-to-bead transfer with bone marrow-derived human mesenchymal stem cells on microcarriers: Utilising the phenomenon to improve culture performance. Biochem. Eng. J..

[61] Schop D., van Dijkhuizen-Radersma R., Borgart E., Janssen F.W., Rozemuller H., Prins H.J., de Bruijn J.D. (2010). Expansion of human mesenchymal stromal cells on microcarriers: Growth and metabolism. J. Tissue Eng. Regen. Med..

[62] Higuera G., Schop D., Janssen F., van Dijkhuizen-Radersma R., van Boxtel T., van Blitterswijk C.A. (2009). Quantifying In Vitro Growth and Metabolism Kinetics of Human Mesenchymal Stem Cells Using a Mathematical Model. Tissue Eng. Part A.

[63] Balducci L., Blasi A., Saldarelli M., Soleti A., Pessina A., Bonomi A., Coccè V., Dossena M., Tosetti V., Ceserani V. (2014). Immortalization of human adipose-derived stromal cells: Production of cell lines with high growth rate, mesenchymal marker expression and capability to secrete high levels of angiogenic factors. Stem Cell Res. Ther..

[64] Leber J., Barekzai J., Blumenstock M., Pospisil B., Salzig D., Czermak P. (2017). Microcarrier choice and bead-to-bead transfer for human mesenchymal stem cells in serum-containing and chemically defined media. Process Biochem..

[65] Yin D., Wells J., Clinton J., Zou C. Comparative analysis of cell proliferation, immunosuppressive action, and multi-lineage differentiation of immortalized MSC and MSC from bone marrow, adipose tissue, and umbilical cord blood. Proceedings of the International Society for Stem Cell Research Conference.

[66] Wolbank S., Stadler G., Peterbauer A., Gillich A., Karbiener M., Streubel B., Wieser M., Katinger H., van Griensven M., Redl H. (2009). Telomerase immortalized human amnion- and adipose-derived mesenchymal stem cells: Maintenance of differentiation and immunomodulatory characteristics. Tissue Eng. Part A.

[67] Bourin P., Bunnell B.A., Casteilla L., Dominici M., Katz A.J., March K.L., Redl H., Rubin J.P., Yoshimura K., Gimble J.M. (2013). Stromal cells from the adipose tissue-derived stromal vascular fraction and culture expanded adipose tissue-derived stromal/stem cells: A joint statement of the International Federation for Adipose Therapeutics and Science (IFATS) and the International Society for Cellular Therapy. Cytotherapy.

